# Structural reorganization of the fungal endoplasmic reticulum upon induction of mycotoxin biosynthesis

**DOI:** 10.1038/srep44296

**Published:** 2017-03-13

**Authors:** Marike Johanne Boenisch, Karen Lisa Broz, Samuel Owen Purvine, William Byron Chrisler, Carrie Diana Nicora, Lanelle Reine Connolly, Michael Freitag, Scott Edward Baker, Harold Corby Kistler

**Affiliations:** 1 Department of Agronomy and Plant Genetics, University of Minnesota, St. Paul, MN 55108, USA; 2 USDA ARS Cereal Disease Laboratory, St. Paul, MN 55108, USA; 3 Pacific Northwest National Laboratory, Richland, WA 99354 USA; 4 Department of Biochemistry and Biophysics, Oregon State University, Corvallis, OR 97331, USA; 5 Department of Plant Pathology, University of Minnesota, St. Paul, MN 55108, USA

## Abstract

Compartmentalization of metabolic pathways to particular organelles is a hallmark of eukaryotic cells. Knowledge of the development of organelles and attendant pathways under different metabolic states has been advanced by live cell imaging and organelle specific analysis. Nevertheless, relatively few studies have addressed the cellular localization of pathways for synthesis of fungal secondary metabolites, despite their importance as bioactive compounds with significance to medicine and agriculture. When triggered to produce sesquiterpene (trichothecene) mycotoxins, the endoplasmic reticulum (ER) of the phytopathogenic fungus *Fusarium graminearum* is reorganized both *in vitro* and *in planta*. Trichothecene biosynthetic enzymes accumulate in organized smooth ER with pronounced expansion at perinuclear- and peripheral positions. Fluorescence tagged trichothecene biosynthetic proteins co-localize with the modified ER as confirmed by co-fluorescence and co-purification with known ER proteins. We hypothesize that changes to the fungal ER represent a conserved process in specialized eukaryotic cells such as in mammalian hepatocytes and B-cells.

Filamentous fungi produce a remarkable diversity of low molecular mass natural products with unique bioactive properties. Many of these have been exploited as pharmaceuticals and antibiotics while others have negative impacts as mycotoxins^1^,^2^. Whole genome sequencing has revealed fungal genomes to be rich sources of such secondary metabolites, including polyketides, terpenoids, alkaloids or small peptides, often encoded as part of large biosynthetic gene clusters. However, attempts to characterize and exploit the products of these gene clusters by expression in heterologous systems have met with limited success^3^. It has been suggested that, in addition to the biosynthetic enzymes themselves, their spatial organization within the cell may be adapted for efficient metabolite biosynthesis. Nevertheless, cellular compartmentalization of secondary metabolic pathways in filamentous fungi is poorly characterized^4^,^5^.

*Fusarium graminearum* is a pathogenic mold that contaminates wheat and barley crops with sesquiterpene, trichothecene (TRI) mycotoxins. These metabolites are harmful through acute and chronic exposure^6^,^7^; and may cause hemorrhaging, diarrhea, emesis, weight loss, immunomodulation and death^8^,^9^. TRIs also are efficient inhibitors of eukaryotic protein synthesis^10^,^11^. Most developed nations set maximum limits for TRI allowable in food products^12^.

TRI biosynthesis begins with the cyclization of farnesyl pyrophosphate, catalyzed by the enzyme trichodiene synthase Tri5 (Fig. 1). The sesquiterpene product trichodiene is progressively modified, mostly through hydroxylation, by cytochrome P450 monoxygenases including trichodiene oxygenase (Tri4) and calonectrin oxygenase (Tri1)^14^,^15^. TRIs produced by *F. graminearum* include deoxynivalenol (DON) and acetylated derivatives such as 15-acetyl-DON (15-ADON). Fluorescent protein-tagged enzymes Tri1 and Tri4 co-localize to ~3 μm structures that were provisionally called “toxisomes”^16^. Upon induction of TRI synthesis, the mevalonate pathway enzyme 3-hydroxy-3-methylglutaryl-CoA reductase (HMG-CoA reductase or Hmr1) also co-localizes with Tri4 at toxisomes^16^.

Here we characterize the subcellular changes that occur upon induction of the TRI products in fungal cells. We discovered that *F. graminearum* toxisomes are proliferations of the smooth endoplasmic reticulum (SER), and that changes in ER organization during mycotoxin production resemble those occurring during induction of drug-detoxifying cytochrome P450 monoxygenases in mammalian hepatocytes.

## Results

Structural changes of the ER in *F. graminearum* were studied using reporter strains expressing fluorescence tagged proteins (Table 1). Schemes detailing tagging strategies and validations for transformants are provided (Supplementary Fig. S1; primer sequences in Supplementary Table S1).

### The enzyme Hmr1 is localized at the ER and indicates ER reorganization upon TRI induction

**Figure 1:**
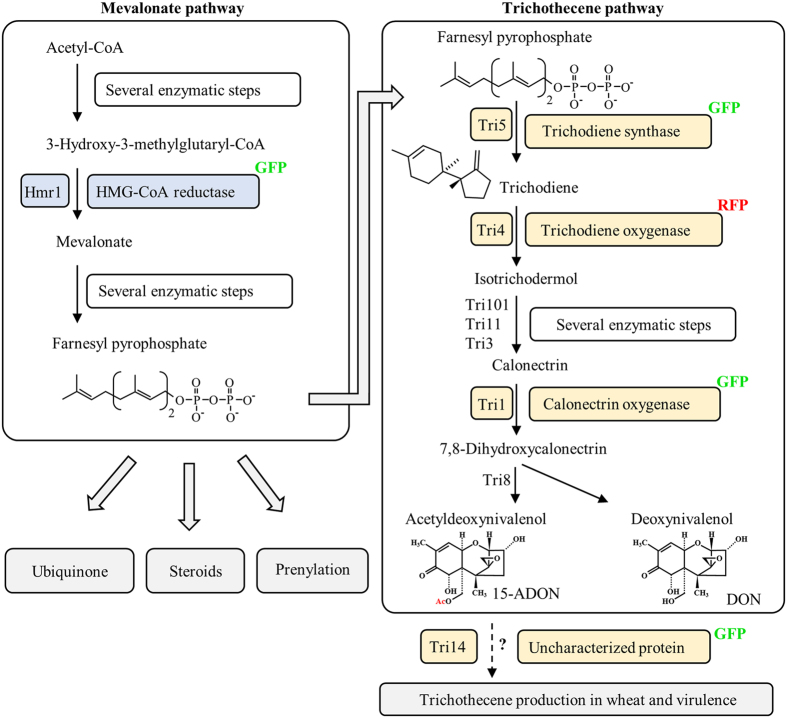
Primary and secondary metabolism pathways in *F. graminearum* and fluorescence tagged proteins. The mevalonate pathway enzyme HMG-CoA reductase (Hmr1), and the trichothecene biosynthetic pathway enzymes trichodiene synthase (Tri5), trichodiene oxygenase (Tri4), calonectrin oxygenase (Tri1), as well as Tri14 were tagged at the C-terminus with fluorescent proteins RFP or GFP. Hmr1 catalyzes the synthesis of mevalonate, leading to farnesyl pyrophosphate (FPP), which is utilized by essential metabolic pathways (e.g. biosynthesis of ubiquinone, steroids and terpenes) as well as the trichothecene pathway. After cyclization of FPP by Tri5, oxygenation of the trichodiene product by Tri4 creates the toxic epoxide moiety in isotrichodermol and subsequent pathway intermediates. Further reactions catalyzed by Tri101, Tri11, Tri3, Tri1, and Tri8 generate deoxynivalenol (DON) and 15- acetyldeoxynivalenol (15-ADON). The reason why Tri14 is required for DON synthesis *in planta* is not known^13^.

**Figure 2:**
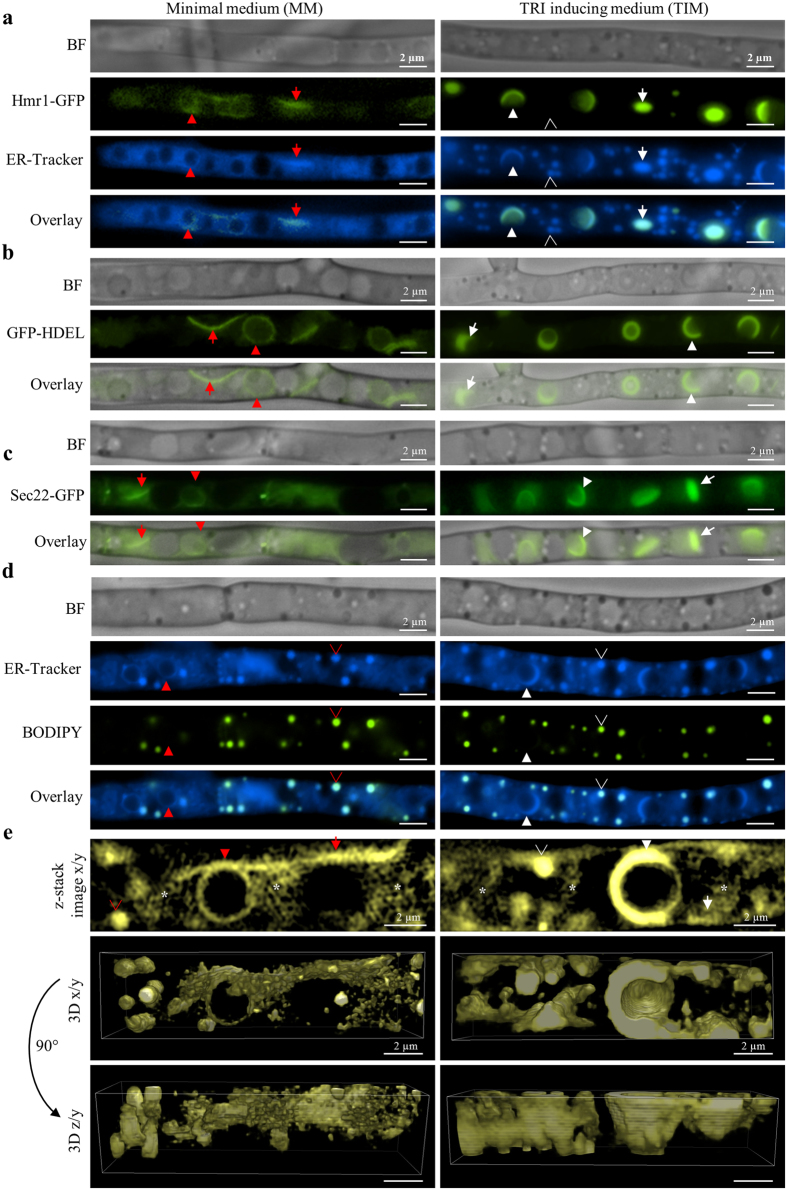
Reorganization of the ER upon TRI production. (**a–e)** Reporter strains Hmr1-GFP (**a**), GFP-HDEL (**b**), Sec22-GFP (**c**) as well as the wild type PH-1 (**d** and **e**) were grown in MM (left panels) or TIM (right panels). (**a)** Fluorescence of Hmr1-GFP and ER-Tracker co-localize at reticulate peripheral ER (red arrow) and circular perinuclear ER (red arrowhead) in MM, and at ovoid structures (white arrow) and crescent structures (white arrowhead) upon TRI induction. Smaller circular structures (unfilled white arrowhead) stained with ER-Tracker, do not co-localize with Hmr1-GFP and were identified as lipid bodies (see **d**). (**b** and **c**) Similar differences in ER pattern in MM or TIM are observed with GFP-HDEL (**b**), Sec22-GFP (**c**) and with the wild type PH-1 stained with ER-Tracker (**d**). (**d)** Dual staining of PH-1 with ER-Tracker and BODIPY shows that lipid bodies (unfilled arrowheads) co-localize with ER-Tracker in MM and TIM. (**e**) Super resolution microscopy of the ER of PH-1 stained with ER-Tracker (yellow) in MM or TIM. Single images from z-stacks of the ER (row 1) and 3D reconstruction (row 2 x/y view, and 3 y/z view; Supplementary Movies S1, S2) demonstrate the reticulate pattern of the perinuclear ER (red arrowhead) and peripheral ER (red arrow) in MM and the thickened perinuclear ER (white arrowhead) and peripheral ER (white arrow) in TIM in detail. The focal x/y view of a z-stack image shows a fine network of ER tubules and vesicular structures. This fine network (asterisk) seems to be connected to other parts of the ER as well as to lipid bodies (unfilled arrowheads).

Hmr1 is a key enzyme in the isoprenoid biosynthetic pathway, catalyzing the rate limiting step in the synthesis of farnesyl pyrophosphate, the initial substrate for TRI biosynthesis (Fig. 1). Hmr1 is an integral membrane protein of the ER in yeast^17^, plants^18^, and animals^19^,^20^ and regulated at the transcriptional, translational, and post-translational level^21^. In *F. graminearum* the expression of Hmr1 is highly upregulated during TRI induction *in vitro* and *in planta*^22^,^23^. To test whether Hmr1 in *F. graminearum* is also localized at the ER, a GFP (green fluorescent protein) tagged strain Hmr1-GFP was grown in minimal medium (MM) (Fig. 2a, left panels) or in a TRI inducing medium (TIM) (Fig. 2a, right panels) for 48 h and stained with the fluorescent dye ER-Tracker Blue-White DPX (named ER-Tracker hereafter). Bright field microscopy (BF) and fluorescence microscopy (FM) revealed that Hmr1-GFP (Fig. 2a, row 2) and ER-Tracker (Fig. 2a, row 3) co-localize both in cells grown in MM and in TIM (Fig. 2a, row 4). In MM, fluorescence of Hmr1-GFP is faint and observed at thin spherical and reticulate peripheral structures, which co-localize with ER-Tracker (Fig. 2a, red arrows and arrowheads). Upon TRI induction, Hmr1-GFP fluorescence has a higher intensity at thickened spherical and crescent structures, as well as ovoid structures, which co-localize with ER-Tracker (Fig. 2a, white arrows and arrowheads). The fluorescence pattern observed under TIM and MM are consistent with previous observations of Hmr1-GFP in *F. graminearum*^16^,^23^. This indicates that Hmr1 is localized to the ER under both conditions and that reorganization of the ER may be occurring upon TRI induction.

### Additional ER markers confirm the reorganization of the ER upon TRI induction

To test if the reorganization of the ER may be affected by ER-Tracker or the staining procedure, ER structure was visualized under similar conditions using strains expressing GFP linked to other ER targeted proteins. The first approach was to constitutively express GFP fused at the carboxyl terminus with the four amino acid ER retention signal HDEL. GFP-HDEL is a known marker of the ER lumen^24^. The *F. graminearum* GFP-HDEL strain visualizes the ER network without ER-Tracker staining (Fig. 2b). By BF and FM of the GFP-HDEL strain, changes in ER structure in hyphae grown in MM (Fig. 2b, left) compared to TIM (Fig. 2b, right) were visible and similar to those observed with Hmr1-GFP. A thin circular, presumably perinuclear ER and reticulate peripheral ER was observed in hyphae grown in MM, while thicker crescent, spherical, and ovoid structures were visible in cells grown in TIM (Fig. 2b, row 2). Circular and crescent structures are shown to be perinuclear ER below using a H4-GFP/Tri4-RFP tagged strain.

A second approach to visualize changes occurring in ER structure was to GFP tag the endogenous vesicle SNARE (soluble N-ethylmaleimide-sensitive-factor attachment receptor) protein Sec22. Sec22 is localized at the ER in *Aspergillus oryzae*^25^, animals, and yeast^26^,^27^. GFP fluorescence in hyphae grown in MM (Fig. 2c, left) and TIM was observed by BF and FM (Fig. 2c, right). A thin perinuclear and reticulate peripheral ER was observed in hyphae of the Sec22-GFP strain grown in MM, while thicker crescent, spheres, and ovoid structures were observed in TIM (Fig. 2c, row 2). Occasionally, GFP fluorescence of both HDEL-GFP and Sec22-GFP strains was observed at vesicles or as a diffuse background in the cytoplasm. We conclude that the differences in ER structure observed in MM compared to TIM are not caused by ER-Tracker staining, since HDEL-GFP and Sec22-GFP strains show the same ER reorganization without ER-Tracker.

### ER reorganization upon TRI induction also occurs in the wild type

To confirm that reorganization of the ER occurs in the absence of fluorescent proteins, the ER of the wild type *F. graminearum* strain PH-1 was stained with ER-Tracker and examined by BF and FM after 48 h in MM or TIM (Fig. 2d). The characteristic thin reticulate ER in MM and tubular, thickened ER in TIM were observed by ER-Tracker (Fig. 2d, row 2) as described with GFP tagged strains above. However, comparing the ER pattern observed with ER-Tracker and fluorescently tagged ER marker proteins (Hmr1-GFP, GFP-HDEL, and Sec22-GFP) suggests that lipid bodies (LB) are stained with ER-Tracker, but were not detected with any of the ER marker proteins (Fig. 2a–c). LB were identified in PH-1 grown in MM (Fig. 2d, left) and TIM (Fig. 2d, right) with the LB specific, green fluorescent dye BODIPY 493/503 (called BODIPY thereafter) (Fig. 2d, row 3) and with ER-Tracker (Fig. 2d, row 2). Co-localization of BODIPY and ER-Tracker was observed in MM and TIM (Fig. 2d, row 4). The diameter of LB were measured in a z-stack of hyphae grown in MM or TIM (n = 92 each) (data not shown). The mean LB diameter was ~0.6 μm but had a broad size distribution ranging between ~0.2 and 1.2 μm. Since LB emerge from, and may be connected to the ER^28^,^29^, the hydrophobic dye ER-Tracker might be transported through the ER to LB, while proteins GFP-HDEL and Sec22-GFP are not. In summary, we infer that ER-Tracker reveals the native structure of the ER in MM and TIM and additionally identifies LB that may be connected with and derived from the ER.

In order to visualize fine structures of the ER < 200 nm, we stained PH-1 with ER-Tracker and acquired z-stacks by super resolution microscopy (SRM) using a Nikon Ti-E microscope equipped with a Nikon structured illumination system. Images from SRM z-stacks and three dimensional (3D) reconstruction of the ER based on the z-stacks confirmed the reorganization of the ER upon TRI induction (Fig. 2e, Supplementary Movie S1, S2) as observed with tagged strains (Fig. 2a–c). Fungal cells grown in MM show a thin perinuclear and peripheral ER (Fig. 2e, left), whereas in TIM a prominent perinuclear and peripheral ER is observed (Fig. 2e, right). The reticulate peripheral ER and the circular perinuclear ER in MM was most distinct in z-stack images at the center of the hyphae (Fig. 2e, row 1). SRM revealed a network of fine ER tubules and vesicles in the cytoplasm in MM as well as in TIM (Fig. 2e, asterisks), but less distinct in the latter. This fine tubular network appears to be connected to perinuclear, peripheral ER, and LB under both conditions.

The 3D view of the reconstructed ER in MM and TIM demonstrates the organization of the ER network in the cell (Fig. 2e, row 2, 3, Supplementary Movie S1, S2). The thin perinuclear and peripheral ER, as well as thin ER tubules and vesicles, can be observed in MM, while an asymmetric thickening of perinuclear ER and pronounced peripheral ER are visible in TIM. Since the characteristic ER patterns observed in MM and TIM with GFP tagged strains (Fig. 2a–c) were also present using ER-Tracker in the wild type PH-1 (Fig. 2d and e), the hypothesis that the observed ER changes occur due to the presence of fluorescence tagged ER proteins can be rejected.

### TRI pathway enzymes Tri4 and Tri1 co-localize at the ER upon TRI induction

**Figure 3:**
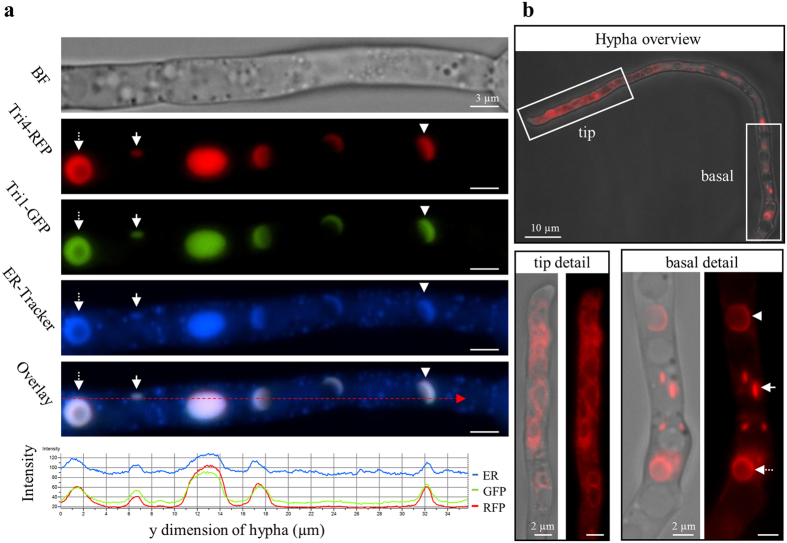
Localization of TRI pathway proteins Tri1 and Tri4 during TRI induction *in vitro*. (**a)** Fluorescence microscopy of strain Tri4-RFP/Tri1-GFP grown in TIM stained with ER-Tracker. Tri4 co-localizes with Tri1 and the ER at spheres (dashed arrow), crescents (arrowheads) and ovoid structures (arrow) but not in lipid bodies (see also Supplementary Fig. S2, Movie S3). Peaks of fluorescence intensity of ER-Tracker, Tri4-RFP and Tri1-GFP occur at all structures described (trace indicated by red dashed arrow in the overlay image). (**b)** After 48 h of growth in TIM, hyphal tips of strain Tri4-RFP exhibit a reticulate fluorescence pattern (overview and tip detail), while in basal parts of hyphae spheres (dashed arrow), crescents (white arrowheads), and ovoid structures (white arrow) are abundant (overview and basal detail).

Experiments were conducted to determine if trichodiene oxygenase (Tri4) and the calonectrin oxygenase (Tri1), enzymes that catalyze early and late steps, respectively, of the TRI biosynthetic pathway in *F. graminearum* (Fig. 1), were localized to the ER during TRI synthesis. ER-Tracker staining of the dual fluorescence tagged strain Tri4-RFP (red fluorescent protein)/Tri1-GFP grown in TIM shows that both proteins localize to the same positions in the cell (Fig. 3a). Both Tri4-RFP and Tri1-GFP were observed at spherical and crescent shaped ER structures, as well as at smaller ovoid structures (Fig. 3a, row 2, 3). Co-localization of both Tri4-RFP and Tri1-GFP with ER-Tracker is shown in the overlay image of the three fluorescence channels (Fig. 3a, row 2–5) and by an intensity profile of the fluorescence channels along the hypha (Fig. 3a, row 6). No co-localization of Tri4-RFP to LB stained with ER-Tracker and BODIPY was observed (Supplementary Fig. S2, Movie S3). In summary, both Tri1 and Tri4 co-localize with ER-Tracker, and given that Hmr1-GFP has been shown to co-localize with Tri4-RFP^16^ and ER-Tracker in TIM (Fig. 2a), we infer that the three enzymes co-localize at the ER.

### Spatial and temporal changes of the ER upon TRI induction

As previously observed, TRI induction precipitates cellular changes such as hyphal swelling that often first occur behind the advancing hyphal tip cell^30^. These subapical cells also show ER modifications in TRI induced hyphae. Within Tri4-RFP hyphae grown in TIM for 48 h, subapical cells show spherical, crescent, and smaller ovoid structures while the hyphal tip cells exhibit a thin reticulate ER (Fig. 3b). This observation was also made with Tri1-GFP and dual tagged strains (Tri4-RFP/Tri1-GFP) under similar conditions (data not shown). The reticulate ER pattern of hyphal tips in TIM is similar to the ER pattern observed under non-inducing conditions in MM with Hmr1-GFP, ER-Tracker, GFP-HDEL, and Sec22-GFP strains (Fig. 2a–c, left). Early in the process of induction, when hyphae of the Tri1-GFP strain were grown for only 20–24 h in TIM, we also observed faint reticulate GFP pattern of the ER in tip cells and basal parts of hyphae (data not shown). In summary, the results show that the ER of *F. graminearum* undergoes a distinct spatial and temporal maturation process during TRI induction and that tagged proteins Tri4-RFP, Tri1-GFP, and Hmr1-GFP are localized to the ER as they are expressed, whether the ER is modified or not.

### ER membranes are enriched for TRI pathway proteins and conserved ER proteins

**Figure 4:**
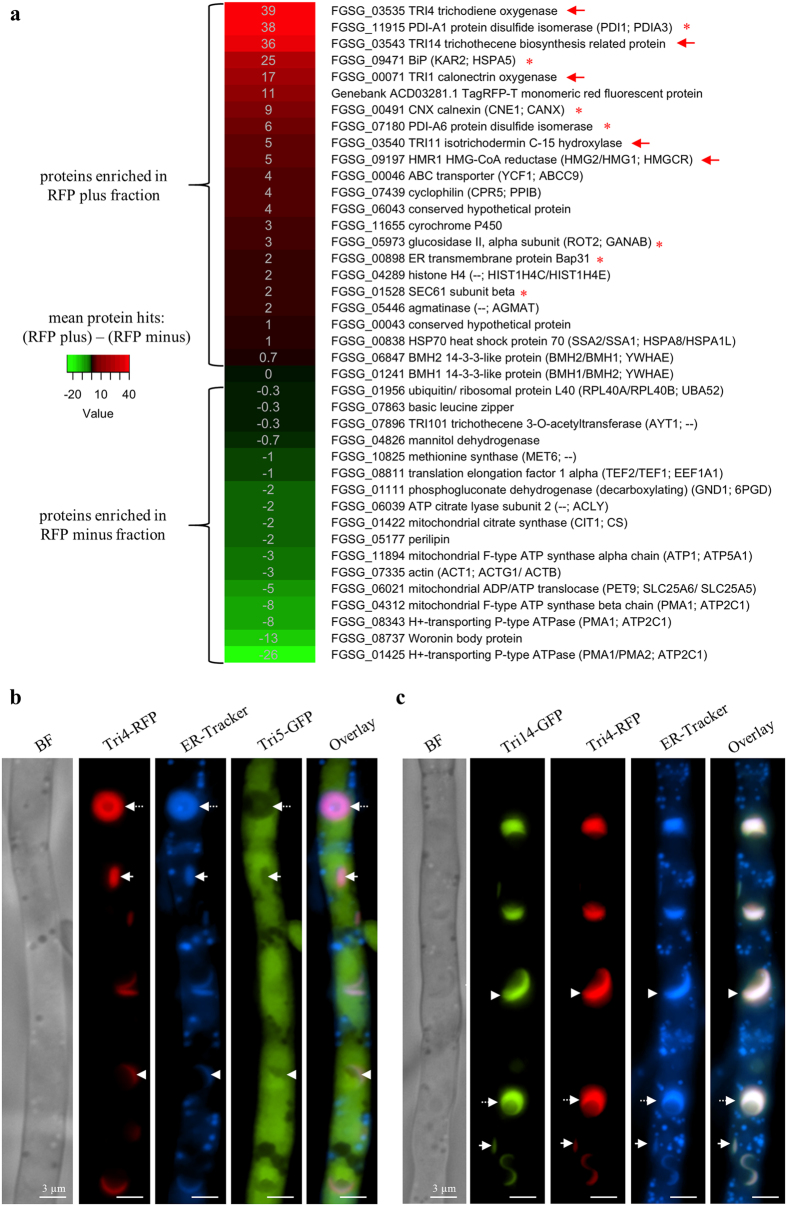
Proteome of Tri4-RFP-associated membranes (**a**) and cellular localization of Tri5-GFP (**b**) and Tri14-GFP (**c**). (**a)** Heat map showing proteins of greater abundance in “RFP plus” (red) or “RFP minus” (green) fractions (see also Supplementary Fig. S3 and Supplementary Dataset S1 and S2). From left to right: Heat map of proteins, gene IDs (FGSG_#), predicted function, and predicted orthologs from (*Saccharomyces cerevisiae*; *Homo sapiens*) in brackets (-- no homolog) (see also Supplementary Dataset S2). Four Tri proteins (Tri4, Tri14, Tri1, Tri11), Hmr1 (red arrow), and ER related proteins (red asterisk) are the most abundant proteins in RFP plus fractions, while proteins associated with other membranous organelles or the cytoplasm are enriched in the RFP minus fraction (see also Supplementary Fig. S4). **(b** and **c)** Fluorescence microscopy of strain Tri5-GFP/Tri4-RFP (**b**) and Tri14-GFP/Tri4-RFP (**c**) grown 48 h in TIM and stained with ER-Tracker. Fluorescence of Tri4-RFP is visible at spheres (black arrow), crescents (white arrowheads), and ovoid structures (white arrow), which co-localize with ER-Tracker in both strains (**c** and **d**). Tri5-GFP fluorescence is observed in the cytosol and later also in vacuoles (see Supplementary Fig. S5), whereas Tri14-GFP co-localizes with Tri4-RFP at the ER.

ER membranes formed in a Tri4-RFP tagged strain after 48 h in TIM were enriched from cellular lysates of protoplasts using Fluorescence-Activated Cell Sorting (FACS) and proteomic analysis was performed (Fig. 4a). Particles in the lysate were assessed for RFP fluorescence intensity and size (Supplementary Fig. S3). Similar size particles differing in RFP fluorescence intensity were sorted into “RFP plus” and “RFP minus” fractions (Supplementary Fig. S3). Extracted and digested peptides of both fractions from three biological replicates were identified by mass spectrometry and comparative proteomics (Fig. 4a, Supplementary Dataset S1 and S2). TRI biosynthetic enzymes as well as conserved ER proteins were enriched in the RFP plus fraction (Fig. 4a, marked with red arrow or asterisk). Of the top ten peptide hits in the RFP plus fraction, three were the proteins previously identified as associated with the modified ER membrane by protein tagging and fluorescence microscopy: Tri4, Tri1 (Fig. 3), and Hmr1 (Fig. 2a). The fluorescence tag RFP itself was also present. Two other TRI related proteins were enriched in the RFP-plus proteome: Tri14 and Tri11 (Fig. 4a). Tri14 is a protein of unknown function whereas Tri11 is an additional cytochrome P450 oxygenase in the TRI pathway. The remaining top protein hits, as well as other peptides in the RFP plus fraction, matched conserved ER proteins, including BiP, calnexin, and two protein disulfide isomerases (Fig. 4a, asterisks). In fact, 7 of 21 enriched proteins in the RFP plus fraction correspond to mostly luminal ER proteins involved in protein processing (Fig. 4a, asterisks, Supplementary Fig. S4). It should be noted that only four (PDI-A1 protein disulfide isomerase (FGSG_11915), BiP (FGSG_09471), PDI-A6 protein disulfide isomerase (FGSG_07180), and agmatinase (FGSG_05446)) of the 21 enriched proteins have the HDEL or KDEL ER retention signal in the protein sequence (see Supplementary Dataset S2, column I).

Proteins enriched in the RFP minus fraction were abundant cytosolic or membrane associated proteins at the plasma membrane, vacuole, mitochondria or Woronin bodies (Fig. 4a).

### The TRI pathway enzyme Tri5 is localized in the cytosol

One of the enzymes of the TRI biosynthetic pathway missing from the proteomics data was trichodiene synthase (Tri5), which catalyzes the initial cyclization of farnesyl pyrophosphate. To determine whether this enzyme is also localized to the modified ER upon TRI induction, a Tri5-GFP tagged strain was generated in a Tri4-RFP background. The resulting Tri4-RFP/Tri5-GFP dual tagged strain was grown in TIM (Fig. 4b). After 48 h toxin-producing hyphae exhibit fluorescence of Tri4-RFP at spherical, crescent, and ovoid structures, which co-localize with ER-Tracker, while Tri5-GFP fluorescence was visible in the cytosol. Occasionally after 48 h, but more frequently after 72 h, we observed GFP fluorescence also in the lumen of vacuoles, as noted previously^31^. This observation was verified by staining hyphae after 72 h in TIM and MM with the fluorescent vacuolar dye CMAC (Supplementary Fig. S5).

### The TRI pathway associated protein Tri14 co-localizes with Tri4 at the ER

Since proteomic data suggested that Tri14 may be a component of modified ER in TRI induced cells (Fig. 4a), we tagged this uncharacterized protein with GFP to determine its subcellular localization during TRI induction (Fig. 4c). Tri14 is encoded within the TRI biosynthetic gene cluster, is co-regulated with other TRI genes, and its predicted amino acid sequence shows no similarity with proteins in public databases, although it has been proposed to play a role in TRI regulation^13^. BF and FM of hyphae of a Tri14-GFP/Tri4-RFP dual tagged strain grown in TIM revealed that Tri14-GFP co-localized with Tri4-RFP and ER-Tracker at crescent, spherical, and ovoid structures.

### ER modifications are structurally similar *in vitro* and *in planta*

**Figure 5:**
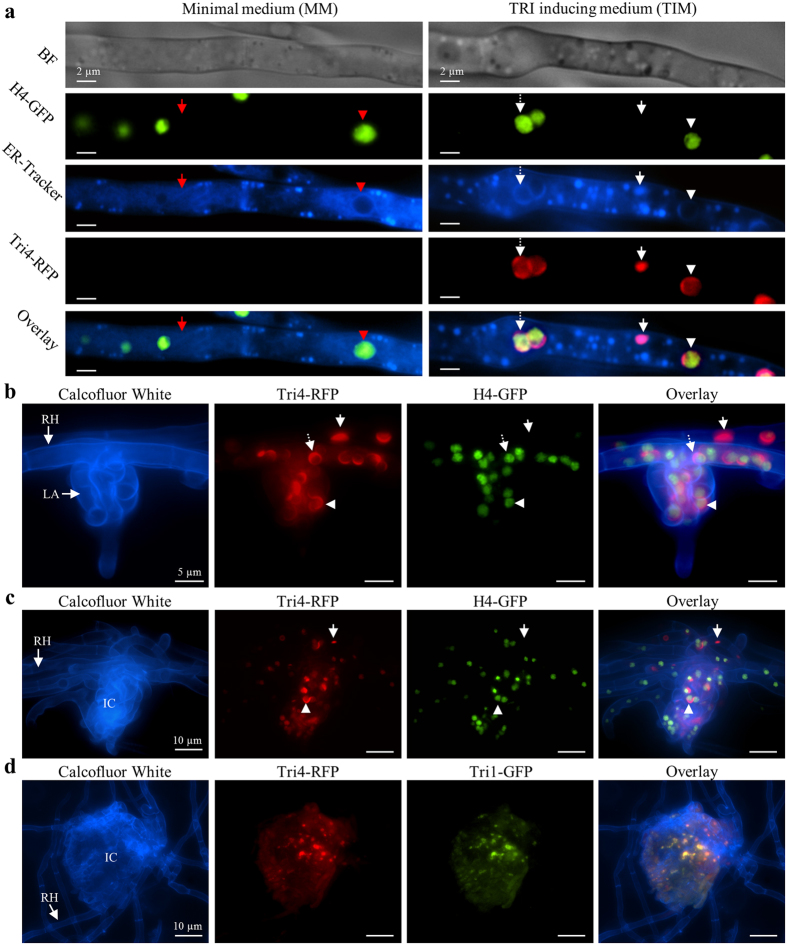
Localization the ER and nuclei *in vitro* (**a**) and *in planta* (**b–d**). (**a–c)** Nuclei and Tri4 visualized with strain H4-GFP/Tri4-RFP *in vitro* (**a**, see also Supplementary Fig. S6) and in plant infection structures including lobate appressoria (**b**) and infection cushions (**c** and **d**). (**a)** Fluorescence of H4-GFP and ER-Tracker are visible in both MM (left column) and TIM (right column). In MM, thin circular structures (red arrowhead) visible by ER-Tracker surround nuclei and thus are perinuclear ER. Reticulate strings (red arrow) not associated with nuclei indicate peripheral ER. In TIM, spheres (dashed white arrow) and crescents (white arrowhead) are visible by ER-Tracker and Tri4-RFP fluorescence and circumscribe nuclei, indicating modified perinuclear ER. Ovoid structures (white arrows) are not spatially associated with nuclei, indicating modified peripheral ER. (**b** and **c)** Infection structures of H4-GFP/Tri4-RFP on a palea of wheat 9 days post inoculation stained with Calcofluor White (blue). (**b**) Runner hyphae (RH) and a lobate appressorium (LA) show crescent structures (arrowheads) and spheres (dashed arrow) by Tri4-RFP, which surround nuclei (green), while ovoid structures (arrows) are not associated to nuclei. (**c)** Observations similar to those described in b for an infection cushion (IC). (**d)** The TRI pathway enzymes Tri4 and Tri1 of a Tri4-RFP/Tri1-GFP strain co-localize at modified ER in an infection cushion 9 days post inoculation.

In order to test whether crescent and spherical structures are associated with the nucleus, we visualized nuclei by tagging a predicted histone H4 coding gene *FghH4-2* of *F. graminearum* (see also Table 1) in a Tri4-RFP tagged strain (Fig. 5a–c). The H4-GFP/Tri4-RFP dual tagged strain was grown in MM (Fig. 5a, left) or TIM (Fig. 5a, right) and stained with ER-Tracker (Fig. 5a, row 3). As anticipated, nuclei labeled by H4-GFP were detected in both media, whereas Tri4-RFP fluorescence was detected only in TIM. In hyphae grown in MM, circular, but not reticulate, structures of the ER circumscribe nuclei (Fig. 5a red arrows and arrowheads). Crescent structures and spheres visible in TIM with ER-Tracker and with Tri4-RFP also delineate nuclei, while ovoid structures do not (Fig. 5a white arrows and arrowheads). Thus, crescents and spheres may be modified perinuclear ER, whereas smaller ovoid structures may be modified peripheral ER. To assure that modified ER is not undergoing autophagy, vacuoles of the H4-GFP/Tri4-RFP strain grown in TIM were stained with the vacuolar lumen dye CMAC and, indeed the modified ER did not co-localize with vacuoles (Supplementary Fig. S6).

To test whether ER modifications occur *in vitro* on the natural plant host, we examined infection structures of *F. graminearum*, which are induced in TRI production during infection of wheat (Boenisch and Schäfer^32^). Point inoculation of paleas and glumes of wheat with conidia of H4-GFP/Tri4-RFP demonstrated that modified perinuclear ER and peripheral ER are formed in smaller (~5–15 μm) lobate appressoria (Fig. 5b) and larger (15–50 μm) infection cushions (Fig. 5c) penetrating the plant epidermis. Calcofluor White was used to visualize the fungal cell walls in infection structures, which exhibited crescent and spherical perinuclear ER containing nuclei and ovoid structures without nuclei (Fig. 5b and c).

Infection structures observed in the same bioassay, but using a Tri4-RFP/Tri1-GFP strain, revealed that the enzymes Tri4-RFP and Tri1-GFP co-localize in infection structures, such as an infection cushion (Fig. 5d), as was observed *in vitro* (Fig. 3a).

### TEM reveals ultrastructural changes of the ER upon TRI induction

**Figure 6:**
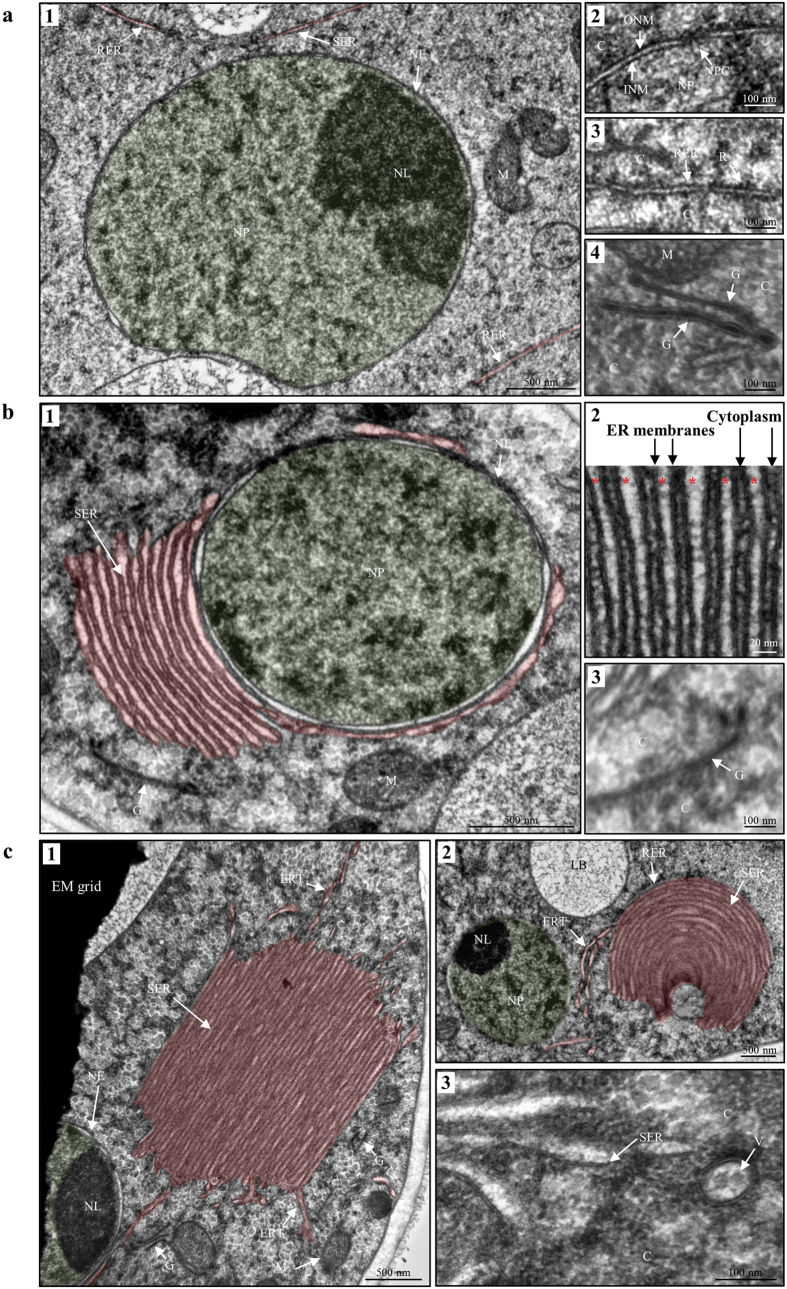
TEM of modified ER. (**a1)** In MM the perinuclear and peripheral ER appear reticulate. The nuclear envelope surrounds the nucleoplasm and the nucleolus. (**a2)** The nuclear envelope consists of the outer and inner nuclear membrane and inserted nuclear pores. (**a3)** Peripheral strings of ER in the cytoplasm show ribosomes, indicating rough ER. (**a4)** Membranous stacks of cisternae with a dark (high osmium) lumen may be Golgi equivalents. (**b1)** Cells grown in TIM show “karmellae”, lamellar stacked smooth ER membranes surrounding the nucleus. (**b2)** Detail image of a karmellae shows the ER lumen (red asterisks) between two ER membranes and the constricted cytoplasmic space. (**b3)** Membranous stacks of cisternae showing a dark lumen are visible in the cytoplasm. (**c1-2)** Lamellar stacks of smooth ER apart from nuclei (“strips”) (**c1**) and concentric stacks of smooth ER (“whorls”) (**c2**) were observed. Fine ER tubules extending from strips and whorls indicate an interconnecting ER network of both smooth and rough ER. (**c3)** Vesicular structures ~90 nm in diameter appear to bud from stacked ER membranes and might be COPII vesicles. The nucleoplasm is colored green and the ER red. Abbreviations: C cytoplasm, ERT ER tubules, G Golgi equivalent, INM inner nuclear membrane, M mitochondrion, NE nuclear envelope, NL nucleolus, NP nucleoplasm, NPC nuclear pore complex, ONM outer nuclear membrane, R ribosomes, RER rough ER, SER smooth ER, V vesicle.

In order to study the ultrastructure of modified ER membranes, high pressure freezing (HPF) and freeze substitution (FS) for transmission electron microscopy (TEM) of hyphae grown in MM (Fig. 6a) or TIM (Fig. 6b and c) was applied. In MM we observed nuclei, containing nucleoplasm and nucleolus, enclosed by a thin nuclear envelope as well as reticulate strings and tubules of rough and smooth ER in the cytoplasm (Fig. 6a1). The usual anatomy of a nuclear envelope with the outer and inner nuclear membrane and nuclear pores was visible (Fig. 6a2). Reticulate ER was observed throughout the cytoplasm with various densities of ribosomes attached (RER) or smooth (SER) (Fig. 6a1 and a3). In the cytoplasm we observed single tubules as well as stacks of 1 to 3 cisternae with a dark (high osmium) lumen, which was separated from the cytoplasm by a membrane (Fig. 6a4). Frequently, we observed cisternae in close proximity to ER or to mitochondria. The tubules and cisternae may be Golgi equivalents, described earlier in hyphae of a related *Fusarium* species using FS for TEM^33^.

In TIM we observed lamellar stacks of SER proliferations attached to nuclei (Fig. 6b1), the so-called “karmellae” membranes^34^. Additionally, other SER proliferations, such as lamellar stacks and concentric stacks of ER membranes apart from nuclei were apparent (Fig. 6c), which previously have been referred to as “strips” and “whorls”^17^,^35^. The term organized smooth ER (OSER)^36^, introduced by Snapp^36^, will be used to refer to all SER proliferations observed in TIM. Karmellae are usually observed at one side of the nucleus (Fig. 6b1), while strips (Fig. 6c1) and whorls are not (Fig. 6c2). OSER membranes show an ER lumen of ~10–20 nm and are stacked with a cytoplasmic space of ~10 nm in diameter (Fig. 6b1 and c2). Dark cisternae in MM (Fig. 6a4) suggested to be Golgi also were observed in TIM (Fig. 6b1 and c1). Thin tubules of RER and SER extending from OSER (Fig. 6c1 and c2) indicate that a fine interconnecting network is present in addition to OSER. It should also be noted that nuclei with a thin nuclear envelope, as observed in MM, were present also in some hyphae grown in TIM (Fig. 6c2). Circular structures ~90 nm in diameter appear to bud from the tips of OSER and presumably could be COPII vesicles (Fig. 6c3).

## Discussion

Using fluorescent protein-tagged TRI enzymes and other ER proteins, combined with proteomics and TEM, we discovered that OSER are formed by the filamentous fungus *F. graminearum* under TRI inducing conditions both *in vitro* and *in planta*. A few dozen ER resident enzymes, such as the mevalonate pathway enzyme Hmr1^17^,^34^,^37^ as well as cytochromes P450 and b5, have been shown to induce ER hypertrophy when expressed at elevated levels in yeast^38^, plants^18^, and animal cells^36^,^39^. Lamellar stacked membranes attached to the nucleus (karmellae), concentric (whorls), and lamellar membrane accumulations (strips) of SER apart from the nucleus observed with *F. graminearum* were described previously in yeast^17^,^34^,^40^. SER proliferations in animal cells^36^,^41^ and plants cells^18^ can additionally exhibit sinusoidal arrays with cubic or hexagonal symmetry^36^,^42^, which have not been observed for yeast or *F. graminearum*. Together, these smooth ER proliferations have been termed OSER^36^. The inducing moiety for ER proliferations of Hmr1 is the multi-spanning C-terminal membrane anchor, since truncated versions with only this moiety were sufficient to induce SER proliferations in various organisms^18^,^37^,^39^,^40^,^42^. The principal ability of proteins inducing OSER seems to be dependent on the sequence elements that determine membrane insertion and retention of OSER inducing proteins, including cytochromes P450, b5 and Hmr1^39^ (and citations within). Changes in the membrane-spanning segment or in an adjacent loop region were shown to significantly influence the morphology of the induced membranes^39^. Attaching fluorescent proteins (YFP or GFP) to the cytoplasmic side of the transmembrane segment of ER resident proteins (Sec61β and Sec61γ subunits, cytochrome P450, and b5) and overexpression in animal cells induced OSER, as resident proteins with native cytoplasmic segments. OSER were absent when cytoplasmic GFP alone was overexpressed or when expressed in the luminal position of Sec61 β^36^. This indicates that the cytoplasmic catalytic activity of ER resident proteins is not necessary for OSER formation. The absence of OSER when monomeric GFP, attached to Sec61 is overexpressed, in contrast to homodimer forming GFP variants expressed at similar levels, indicate that protein-protein-interactions between cytoplasmic portions of resident ER membrane proteins might also be involved in OSER formation^36^. In summary, OSER formation is likely a conserved response to conditions under which intrinsic membrane proteins of the ER (e.g. Hmr1, Sec61, cytochromes P450) are produced at high levels. Consequently, the OSER observed in *F. graminearum* may be a manifestation of this conserved response among eukaryotes, demonstrated here for the first time in filamentous fungi, and including enzymes of an artificially (*in vitro*) or naturally (*in planta*) induced secondary metabolic pathway.

Observation of established ER markers for filamentous fungi, including ER-Tracker^43^, GFP-HDEL^24^, and Sec22-GFP^25^ confirms structural differences of the ER structure in MM and TIM. SRM resolved a fine network of ER tubules (<100 μm) in the cytoplasm under TRI inducing and non-inducing conditions and allowed visualization of the ER in 3D. Fine interconnecting ER tubules were also observed by TEM. ER domains of peripheral ER cisternae and a tubular shaped ER network have been described in yeast by 3D TEM^44^,^45^. Peripheral ER arises from the nuclear envelope as a membrane network of interconnected tubules and cisternae as in other eukaryotes^44^. In MM, where TRI is not induced, the perinuclear and peripheral ER appeared reticulate and thin. Upon TRI induction the ER was strongly thickened as determined with all ER markers used. Sec22-GFP fluorescence was observed occasionally at vesicles, in agreement with observations of Sec22-GFP in *A. oryzae*^25^ and its function in retrograde and anterograde transport between ER and the Golgi^46^,^47^.

Conserved ER proteins, co-purified by FACS with the TRI biosynthetic enzyme Tri4-RFP, were consistent with ER localization inferred from fluorescent labeling of ER proteins and ER-Tracker. Among the ER proteins identified were two predicted protein disulfide isomerases, cyclophilin and the molecular chaperone KAR2/BiP, all predicted to be within the ER lumen and involved in protein maturation. Two predicted ER membrane associated proteins also were enriched in this fraction: calnexin and the transmembrane ER protein Bap31. ER proliferations in yeast are accompanied by an increase in ER resident proteins Kar2 and Sec61^48^, which are involved in unfolded protein response (UPR). The involvement of UPR in ER proliferations is still unclear, however, smooth ER proliferations do not require the UPR. OSER induced by Hmr isozymes neither stimulate nor require the UPR pathway^49^. In both yeast^50^,^51^ and mammals^52^, high expression of cytochrome P450s also causes induction of the UPR, although similarly, UPR is not required for P450-induced ER proliferations.

The top peptide match in the fluorescent fraction of FACS enriched membranes was trichodiene oxygenase, the fluorescently tagged enzyme itself. Other proteins, known to co-localize with trichodiene oxygenase based on co-fluorescence (i.e. Tri1, Hmr1) also were among the top ranked proteins. Tri14, a protein not previously known to be co-localized with trichothecene biosynthetic enzymes, was among the top enriched proteins in the fluorescent fraction and was shown here to co-localize with trichodiene oxygenase by fluorescent protein tagging. mRNA levels of the *Tri14* gene are regulated by the transcription factor Tri6^53^,^54^ and highly upregulated during TRI induction *in vitro* and *in planta*^13^,^55^,^56^. Interestingly, the deletion mutant Δ*Tri14* was dispensable for TRI accumulation *in vitro*, but not *in planta*^13^ prompting the authors to speculate that Tri14 might have a regulatory function for TRI gene expression or TRI transport from the cell *in planta*. Our results are demonstrating Tri14 localization to the ER do not appear to be consistent with these hypotheses.

What is the biological significance of the OSER structures in *F. graminearum*? Because enzyme active sites for cytochrome P450 oxygenases and Hmr1 are predicted to be on the cytoplasmic side of the ER membrane, toxic TRI products and reaction intermediates likely are synthesized in the thin (~10 nm) cytoplasmic spaces between stacked ER membranes of OSER and thus sequestered from ribosomes and mitochondria that are target sites of trichothecene inhibition^10^,^57^. Supplementary Figure S7 illustrates our proposed model of TRI biosynthesis by TRI enzymes in the fungal cell. There also may be spatial and functional specialization of ER compartments, such as membrane turnover and lipid synthesis, or production of secondary metabolites. While proteins in *F. graminearum* that co-purified with TRI biosynthetic enzymes include ER protein maturases, other ER proteins involved in secretion or other cytochrome P450s involved in sterol synthesis were not found. This suggests that neutral lipid synthesis and the canonical protein secretion pathway may be found at distinct, perhaps undifferentiated portions of the ER. In certain animal cells specialized for secretion (e.g. hepatocytes, steroid-synthesizing cells and neurons), the smooth and rough portions of the ER occupy different regions of the cytoplasm indicating not only a functional, but also a spatial separation of RER and SER. ER structure is known to undergo dramatic changes, depending on internal (e.g. cell type, development, metabolic state)^42^ or external factors (e.g. drug exposure, pathogens, disease)^58^,^59^. While the OSER may form in response to the developmental demands of accommodating the abundant accumulation of trichothecene biosynthetic enzymes, the role that the trichothecene toxins themselves may play in this response is currently being addressed experimentally using Tri5 mutants. For fungi, reorganization into OSER and functional compartmentalization of the ER might play an important role in developmental events allowing increased intracellular levels of TRI and TRI pathway enzymes and perhaps other important bioactive fungal pathways and metabolites.

## Methods

All primer sequences are available in Supplementary Table S1. Key reagents, fungal strains, software and their respective sources are provided in Supplementary Table S2. For detailed protocols see Supplementary Methods.

### Plant material and inoculation of wheat

Glumes and paleas of *Triticum aestivum* cultivar Norm were inoculated with *F. graminearum* strains H4-GFP/Tri4-RFP and Tri1-GFP/Tri4-RFP using 20 conidia/μL in 5 μL sterile water as described previously^32^. Microscopy of infection structures was performed between 7 and 10 days post inoculation (dpi) for paleas and 8–13 dpi for glumes.

### Fungal growth conditions

For conidia production the fungus was grown in CMC culture and conidia were harvested as described previously^30^. Liquid minimal medium (MM) and trichothecene inducing medium (TIM) were adapted from a previous source^56^ as described previously^30^.

### Generation of GFP tagged strains

Reporter strains of *F. graminearum* wild type PH-1 (NRRL 31084) expressing GFP (Table 1) were generated using a fusion PCR and protoplast transformation as described earlier^16^. The dual tagged strain H4-GFP/Tri4-RFP was generated by sexual crossing of strain Tri4-RFP and strain H4-GFP.

### Staining procedures

ER staining was done with 20 μM ER-Tracker Blue-White DPX in (2:1) HBSS (Hank’s Balanced Salt Solution) buffer pH 7.2 and MM or TRI inducing medium. Lipid bodies were stained with 0.05 μM BODIPY 493/503. Staining of vacuoles was performed with 100 μM CellTracker Blue dye CMAC (7-amino-4-chloromethylcoumarin) and 1% DMSO as described previously^30^. 0.001% Calcofluor White in PBS buffer pH 7.2 was used to stain fungal cell walls in infection structures.

### Epifluorescence microscopy

Cultures imaged by bright field (BF) or differential interference contrast (DIC) and fluorescence microscopy (FM) were grown for 48 h in MM or TIM. FM was performed using Nikon ECLIPSE90i microscope (Nikon, Melville, USA). A z-stack image is shown in Figs 2a–d, 3a and b (bottom images), 5a (left), and Supplementary Fig. S3a. Maximum intensity projections (MIPs) of z- stack images are shown in Figs 3b (top image), 4b and c, 5a (right), 5b–d, Supplementary Fig. S2, S5 and S6. Bright field (BF) or difference interference contrast (DIC), and overlay images with BF or DIC, show a z-stack image from the center of the respective cell, except for the overlay with BF in Fig. 3b (top image) and Fig. S6 which are MIPs.

### Super resolution microscopy

Super resolution microscopy (SRM) images were acquired using an inverted Nikon Ti-E microscope, equipped with a structured illumination system and an Andor DU-897 X-8444 camera. Six z-stacks with one to three ER-Tracker stained cells were acquired each in MM and TIM. Figure 3e row 1 shows one image of a z-stack from the center of a cell in MM and TIM. 3D reconstructions were done using the shaded surface rendering function of the Nikon NIS elements AR software.

### Transmission electron microscopy

For transmission electron microscopy (TEM) *F. graminearum* strain Tri4-RFP/Tri1-GFP was grown in MM and TIM for 65 h. Cultures were prepared by high pressure freezing (HPF) in a Balzers HPM 010 and freeze substitution (FS) in a Leica AFS2 chamber. Ultrathin microtome sections embedded in epoxy resin Poly/Bed 812 were imaged with microscope FEI Tecnai T12.

### Proteomics of ER membranes

ER membranes formed in a Tri4-RFP tagged strain after 48 h in TIM were enriched from cellular lysates of protoplasts using Fluorescence-Activated Cell Sorting (FACS). Particles in the lysate were assessed for RFP fluorescence intensity and size and sorted into “RFP plus” and “RFP minus” fractions (Supplementary Fig. S3). Extracted and digested peptides of both fractions from three biological replicates were identified by mass spectrometry, MS/MS analysis and comparative proteomics (see also Supplementary Datasets S1 and S2).

## Additional Information

**How to cite this article:** Boenisch, M. J. *et al*. Structural reorganization of the fungal endoplasmic reticulum upon induction of mycotoxin biosynthesis. *Sci. Rep.* **7**, 44296; doi: 10.1038/srep44296 (2017).

**Publisher's note:** Springer Nature remains neutral with regard to jurisdictional claims in published maps and institutional affiliations.

## Supplementary Material

Supplementary Information

Supplementary Movie 1

Supplementary Movie 2

Supplementary Movie 3

Supplementary Dataset S1

Supplementary Dataset S2

## Figures and Tables

**Table 1:** Reporter strains of *F. graminearum* used in this study, gene ID numbers in the FGDB (Fusarium Graminearum Genome Data Base) and NCBI, function or annotation^a^ of tagged proteins, and strain reference Menke^16^^ ^ or this paper^b^.

Strain name (Figure#)	FGDB ID#	NCBI ID#	Function
Hmr1-GFP^16^ (2a)	FGSG_09197	23556160	Primary metabolism
GFP-HDEL^b^ (2b)	Stop and start of FGSG_09471	23556422	No function^a^
Sec22-GFP^b^ (2c)	FGSG_05226	23552416	Vesicle SNARE^a^
Tri4-RFP^16^ (3b)	FGSG_03535	23550838	TRI metabolism
Tri1-GFP/Tri4-RFP^16^ (3a, 5d, 6)	FGSG_00071	23547586	TRI metabolism
Tri5-GFP/Tri4-RFP^b^ (4b)	FGSG_03537	23550840	TRI metabolism
Tri14-GFP/Tri4-RFP^b^ (4c)	FGSG_03543	23550846	TRI metabolism
H4-GFP/Tri4-RFP^b^ (5a–c)	FGSG_05491	23552671	Histone H4^a^
