# Supplementary material for: Structural reorganization of the fungal endoplasmic reticulum upon induction of mycotoxin biosynthesis
Source: Sci Rep. 2017 Mar 13;7:44296. doi: 10.1038/srep44296 (PMC5347122; doi:10.1038/srep44296)
Supplement: Supplementary Information [file srep44296-s1.pdf]

Supplementary Information

Structural reorganization of the fungal endoplasmic reticulum upon induction of mycotoxin biosynthesis

Authors

Marike Johanne Boenisch[1]

Karen Lisa Broz[2]

Samuel Owen Purvine[3]

William Byron Chrisler[3]

Carrie Diana Nicora[3]

Lanelle Reine Connolly[4]

Michael Freitag[4]

Scott Edward Baker[3]

Harold Corby Kistler[2/5]

Affiliations

[1]Department of Agronomy and Plant Genetics, University of Minnesota, St. Paul, MN 55108, USA

[2]USDA ARS Cereal Disease Laboratory, St. Paul, MN 55108, USA

[3]Pacific Northwest National Laboratory, Richland, WA 99354 USA

[4]Department of Biochemistry and Biophysics, Oregon State University, Corvallis, OR 97331, USA

[5]Department of Plant Pathology, University of Minnesota, St. Paul, MN 55108, USA

Contact

Harold Corby Kistler

Email: hckist@umn.edu

Supplementary Information content:

Supplementary Figures S1-S7

Supplementary Movie legends S1-S3

Supplementary Datasets S1 and S2 (available online)

Supplementary Methods

Supplementary Tables S1 and S2

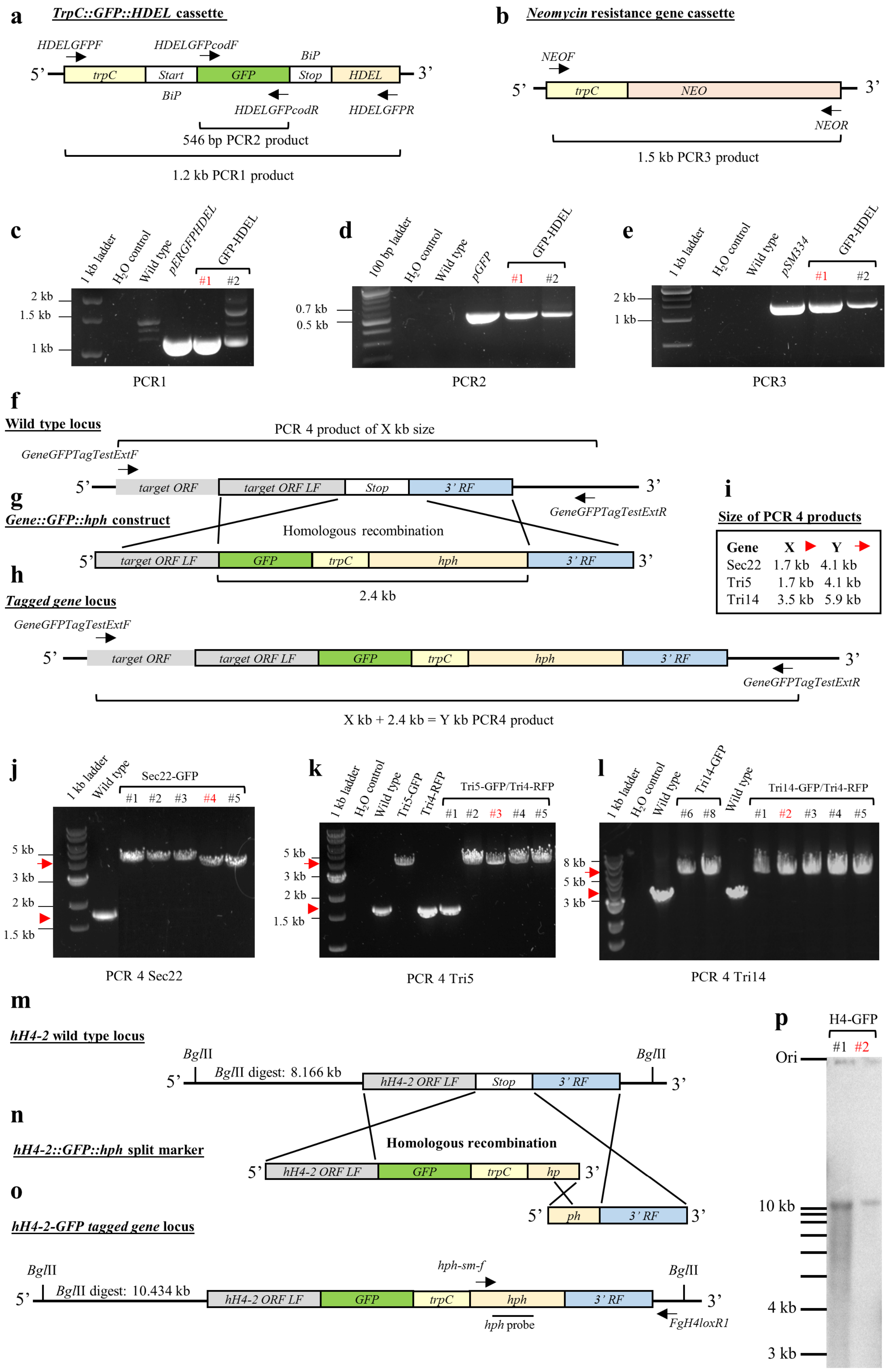
a
TrpC::GFP::HDEL cassette
HDELGFPF
HDELGFPcodF
BiP
5'
trpC
Start
GFP
Stop
HDEL
3'
BiP
HDELGFPcodR
HDELGFPR
546 bp PCR2 product
1.2 kb PCR1 product
b
Neomycin resistance gene cassette
NEOF
5'
trpC
NEO
3'
NEOR
1.5 kb PCR3 product
c
1 kb ladder
H2O control
Wild type
pERGFPHDEL
GFP-HDEL
#1
#2
2 kb
1.5 kb
1 kb
PCR1
d
100 bp ladder
H2O control
Wild type
pGFP
GFP-HDEL
#1
#2
0.7 kb
0.5 kb
PCR2
e
1 kb ladder
H2O control
Wild type
pSM334
GFP-HDEL
#1
#2
2 kb
1 kb
PCR3
f
Wild type locus
PCR 4 product of X kb size
GeneGFPTagTestExtF
5'
target ORF
target ORF LF
Stop
3' RF
3'
GeneGFPTagTestExtR
g
Gene::GFP::hph construct
Homologous recombination
5'
target ORF LF
GFP
trpC
hph
3' RF
3'
2.4 kb
h
Tagged gene locus
GeneGFPTagTestExtF
5'
target ORF
target ORF LF
GFP
trpC
hph
3' RF
3'
GeneGFPTagTestExtR
X kb + 2.4 kb = Y kb PCR4 product
i
Size of PCR 4 products
Gene X Y
Sec22 1.7 kb 4.1 kb
Tri5 1.7 kb 4.1 kb
Tri14 3.5 kb 5.9 kb
j
1 kb ladder
Wild type
Sec22-GFP
#1 #2 #3 #4 #5
5 kb
3 kb
2 kb
1.5 kb
PCR 4 Sec22
k
1 kb ladder
H2O control
Wild type
Tri5-GFP
Tri4-RFP
Tri5-GFP/Tri4-RFP
#1 #2 #3 #4 #5
5 kb
3 kb
2 kb
1.5 kb
PCR 4 Tri5
l
1 kb ladder
H2O control
Wild type
Tri14-GFP
Wild type
Tri14-GFP/Tri4-RFP
#6 #8
#1 #2 #3 #4 #5
8 kb
5 kb
3 kb
PCR 4 Tri14
m
hH4-2 wild type locus
BglII
BglII digest: 8.166 kb
BglII
5'
hH4-2 ORF LF
Stop
3' RF
3'
n
hH4-2::GFP::hph split marker
Homologous recombination
5'
hH4-2 ORF LF
GFP
trpC
hp
3'
5'
ph
3' RF
3'
o
hH4-2-GFP tagged gene locus
BglII
BglII digest: 10.434 kb
5'
hH4-2 ORF LF
GFP
trpC
hph
3' RF
3'
hph-sm-f
BglII
hph probe
FgH4loxR1
p
H4-GFP
#1 #2
Ori
10 kb
4 kb
3 kb

Supplementary Figure S1: Scheme of constructs to generate GFP tagged *F. graminearum* strain GFP-HDEL (a and b), Sec22-GFP, Tri5-GFP/Tri4-RFP and Tri14-GFP/Tri4-RFP strains (f-h) and H4-GFP (m-o) and validation of GFP tagging in transformants GFP-HDEL (c-d), Sec22-GFP (j) Tri5-GFP/Tri4-RFP (k), Tri14-GFP/Tri4-RFP (l) and H4-GFP (p). **a-b** The *trpC::GFP::HDEL* cassette (a), comprising of the *trpC* promoter, the start and stop codon of the predicted *BiP/KAR2* homolog of *F. graminearum*, GFP coding sequence from the plasmid *pGFP::hph::loxP*, and the ER retention signal HDEL was randomly inserted into the genome of the wild type PH-1 by protoplast transformation, along with the neomycin (NEO) resistance gene cassette (b) conferring resistance against neomycin. Binding position of PCR primers (arrow) to validate tagging and expected length of PCR products are illustrated. **c-e** Gel electrophoresis of three different PCR reactions (PCR 1-3 c-e) with specific primer pairs (see also arrows in a and b) to test gDNA of GFP-HDEL transformants #1 and #2 for the *trpC::GFP::HDEL* tagging cassette (c), the GFP coding region (d) and the NEO cassette (e). **c** The *trpC::GFP::HDEL* construct of 1.2 kb is detected with primers *HDELGFPF* and *HDELGFPR* (see also a), using vector *pERGFPHDEL* (positive control) as template in the PCR or gDNA of GFP-HDEL strains #1 and #2, but not using gDNA of wild type PH-1 (lane 3) or water (lane 2). **d** Primers *HDELGFPcodF* and *HDELGFPcodR* bind in the ORF of GFP, amplifying a 600 bp product by PCR (see also a), when the vector *pGFP* (positive control) or gDNA of transformants #1 and 2 is used as template for PCR, but not with water or gDNA of PH-1 (lane 2 and 3). **e** Primers *NEOF* and *NEOR* enable amplification of the NEO cassette of 1.5 kb size (see b), if plasmid pSM334 (positive control) or gDNA of GFP-HDEL #1 and 2 was used as templates for the PCR, but not with gDNA of PH-1 or water. The results from PCR1-3 indicate that the tagging construct and the neomycin resistance cassette were integrated into the genome of both transformants. GFP-HDEL strain # 1 (red font) was used for further studies. **f-i** Scheme for tagging endogenous proteins (Sec22, Tri5 and Tri14) in the *F. graminearum* wild type PH-1 or Tri4-RFP tagged strain (dual tagged) with GFP and validation of strains with primers in PCR4. By homologous recombination between flanking regions upstream (LF) and downstream (RF) of the stop codon of the gene to be tagged (**f**) and homologous flanks of the tagging construct (**g**), the stop codon is replaced by the *GFP::hph* part of the construct, which comprises of the *GFP* coding sequence and hygromycin resistance gene (*hph*) (**h**). Integration of the *GFP::hph* construct in gDNA was tested with gene specific primers (black arrows) binding outside of the flanking regions (**h**) (see also f) and confirmed by a band shift of +2.4 kb compared to the amplicon of PH-1 in PCR 4 (**i**) (see also f-h). **j-l** Gel electrophoresis of PCR products of 5 independent transformants #1-5 of Sec22-GFP (**j**), Tri5-GFP/Tri4-RFP (**k**) and Tri14-GFP/Tri4-RFP (**l**) show a 2.4 kb larger band (red arrow) in transformants compared to the band in wild type or Tri4-RFP samples (red arrowhead). Transformants used for further studies are marked red. **m-o** Scheme for GFP tagging of a predicted histone H4 protein encoded by the *FghH4-2* (FGSG_05491) gene in *F. graminearum* PH-1 (**m**) was similar to genes shown above, however, split-marker fragments (**n**) were used to integrate a 2.3 kb *GFP::hph* construct into the genome (**o**). **p** Correct integration was confirmed in two independent H4-GFP transformants (#1 and #2) by the detection of a 10.434 bp fragment by Southern blotting when a *Bgl*II digest was probed with an *hph* fragment (see *hph* probe in o). Correct integration was also determined by PCR with primers *hph-sm-f* and *FgloxR1* (binds downstream of the 3’ flank used for integration). H4-GFP transformant #2 was used for further studies.

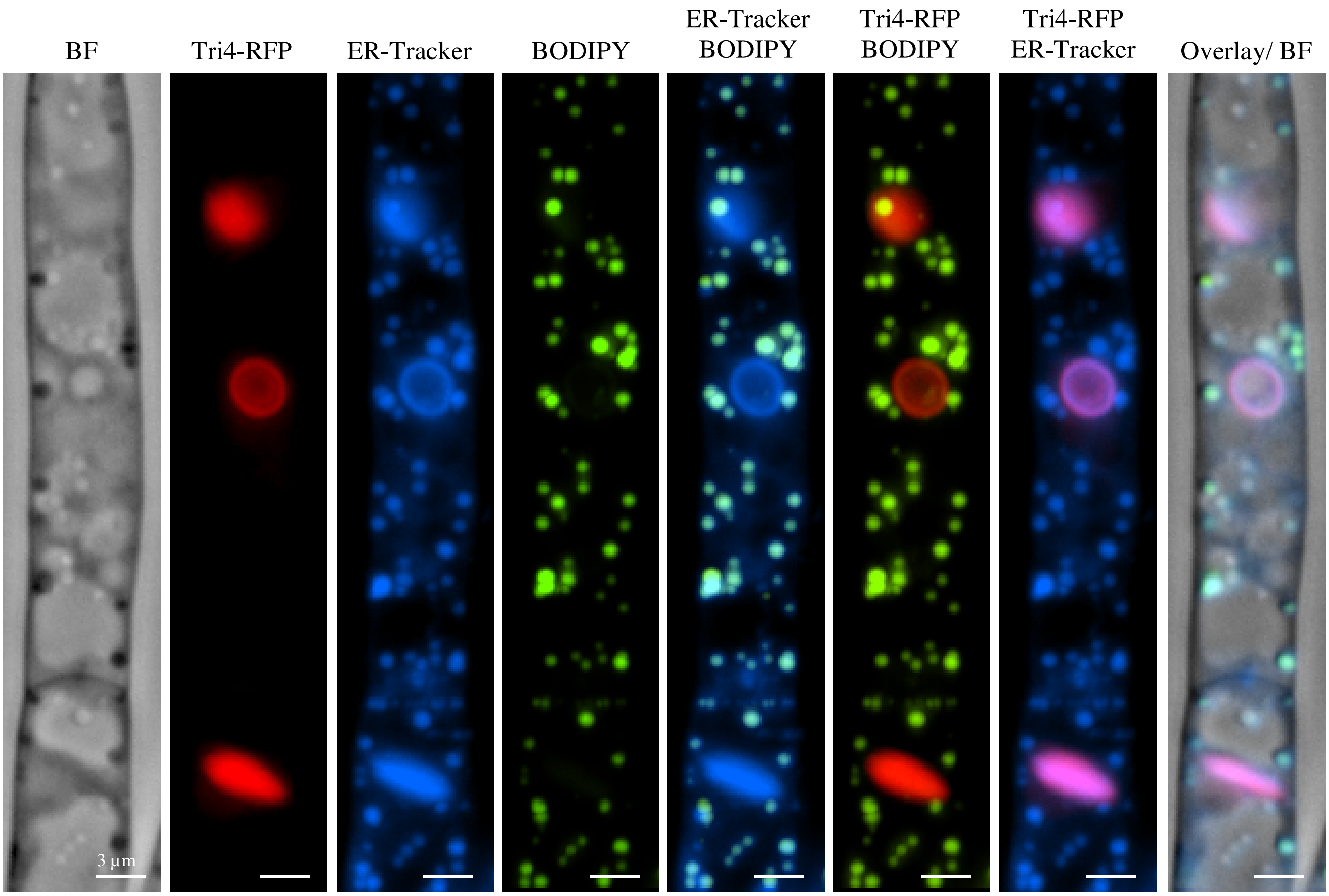

Supplementary Figure S2: Lipid bodies in a Tri4-RFP tagged strain grown in TRI inducing medium for 48 h co-localize with ER-Tracker and the green fluorescent dye BODIPY. Lipid bodies containing neutral lipids are stained by both BODIPY (green) and ER-Tracker (blue) indicating that lipid bodies are connected with the ER and supports their origination from the ER. No co-localization of ER proliferations, visualized by Tri4-RFP and ER-Tracker, and lipid bodies with is observed.

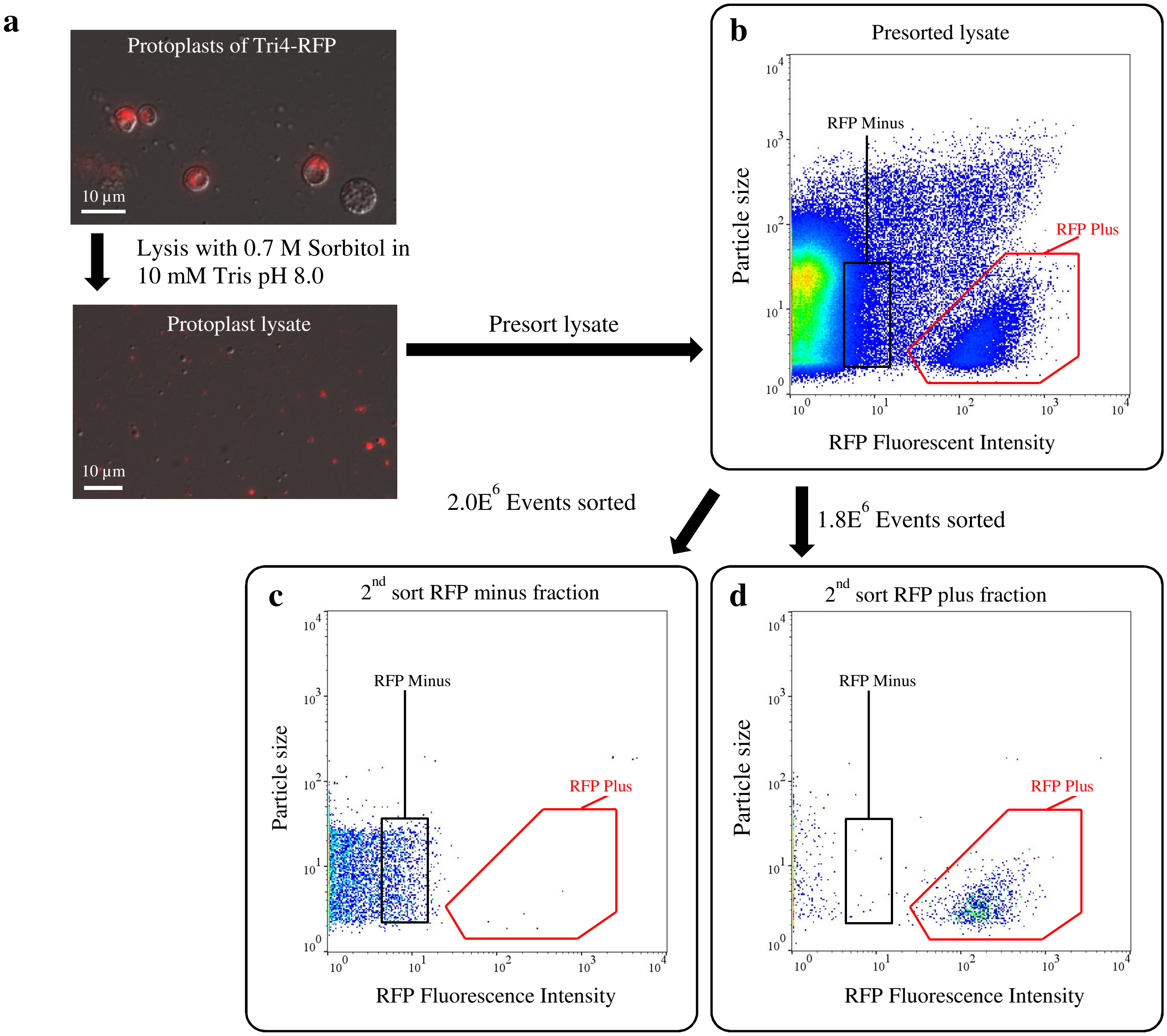

Supplementary Figure S3. Enrichment of modified ER membranes by fluorescence activated cell sorting (FACS). a DIC and RFP overlay image of protoplasts of strain Tri4-RFP with red fluorescent ER proliferations, generated with cell wall degrading enzymes (upper micrograph) and lysed to release red fluorescent ER membranes (bottom micrograph). **b** Particles in the lysate were presorted by FACS with regard to their intensity of RFP fluorescence (x-axis) and size (y-axis) and thereby potential particle aggregates were removed. Particles with the expected size range of modified ER membranes (~3 µm) and a high intensity of RFP fluorescence were enriched in the "RFP plus" fraction (marked area with red outline in b), while particles with similar size, but without RFP fluorescence were enriched in the "RFP minus" fraction (marked area with black outline in b). **c** and **d** By a second sorting step, $2E^{6}$ particles of the RFP minus and $1.8E^{6}$ particles of the RFP plus fractions were further enriched and the resulting RFP minus and RFP plus fractions from the second sorting step (marked areas in c and d) were used for protein extraction and proteomic analysis by LC-MS.

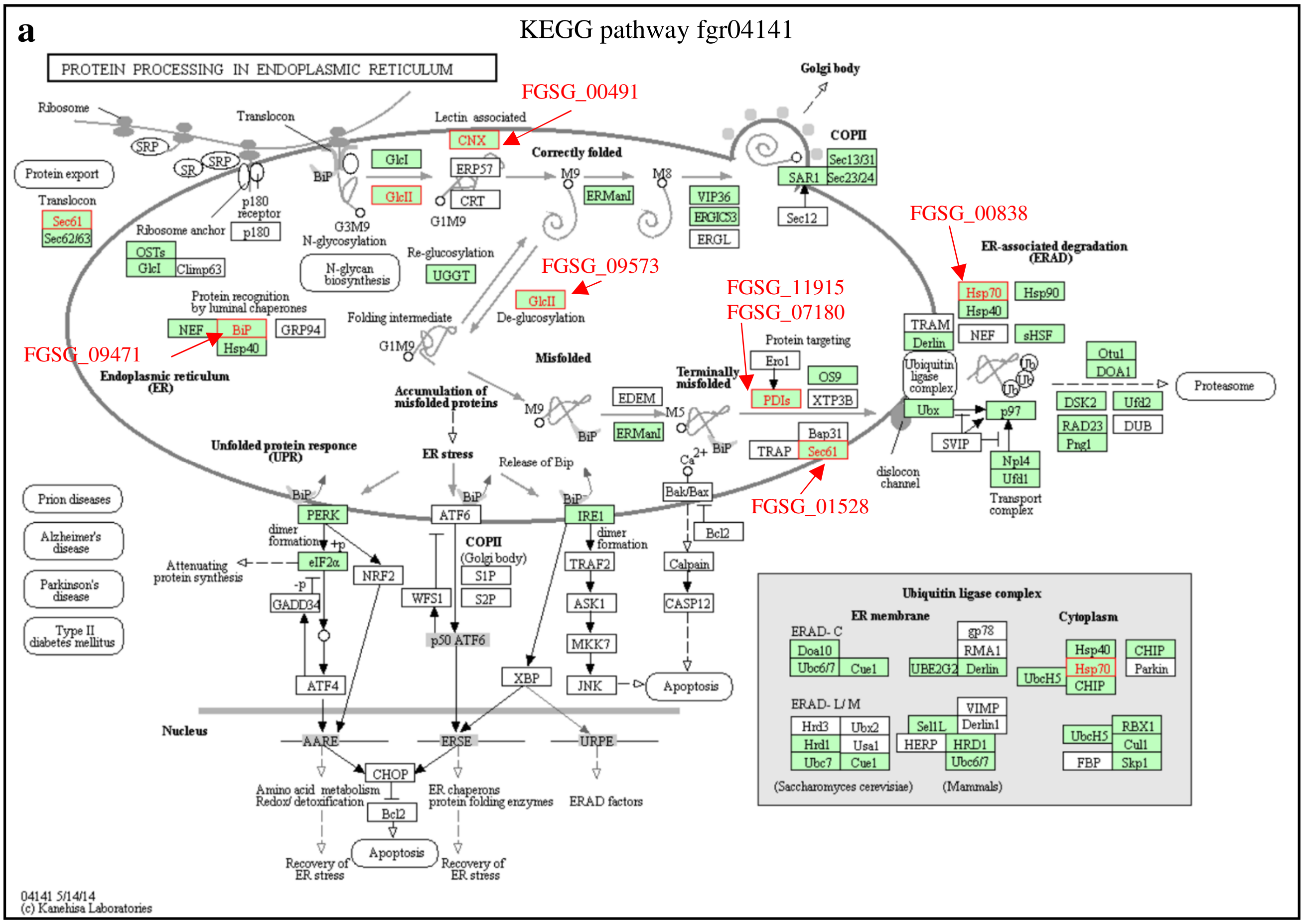

b GenesIDs from RFP plus fraction mapped against KEGG pathway fgr04141

FGSG_03535 FGSG_11915 FGSG_03543
FGSG_09471 FGSG_00071 FGSG_00491
FGSG_07180 FGSG_03540 FGSG_09197
FGSG_00046 FGSG_07439 FGSG_06043
FGSG_11655 FGSG_05973 FGSG_00898
FGSG_04289 FGSG_01528 FGSG_05446
FGSG_00043 FGSG_00838 FGSG_06847

c GenesIDs of protein hits in KEGG pathway fgr04141

FGSG_00491
FGSG_00838
FGSG_01528
FGSG_05973
FGSG_07180
FGSG_09471
FGSG_11915

Supplementary Figure S4: Proteins enriched in RFP plus fraction are involved in protein processing at the ER. a Mapping the top 21 enriched proteins in the RFP plus fraction (**b** and Figure 4a) against the KEGG (Kyoto Encyclopedia of Genes and Genomes)[1] pathway fgr04141 "Protein processing in endoplasmic reticulum" (KEGG data base link http://www.genome.jp/dbget-bin/www_bget?pathway+fgr04141) resulted in 7 protein hits (**c**). Protein hits are marked by red boxes in the pathway and matched gene IDs of *F. graminearum* are given in red fonts.

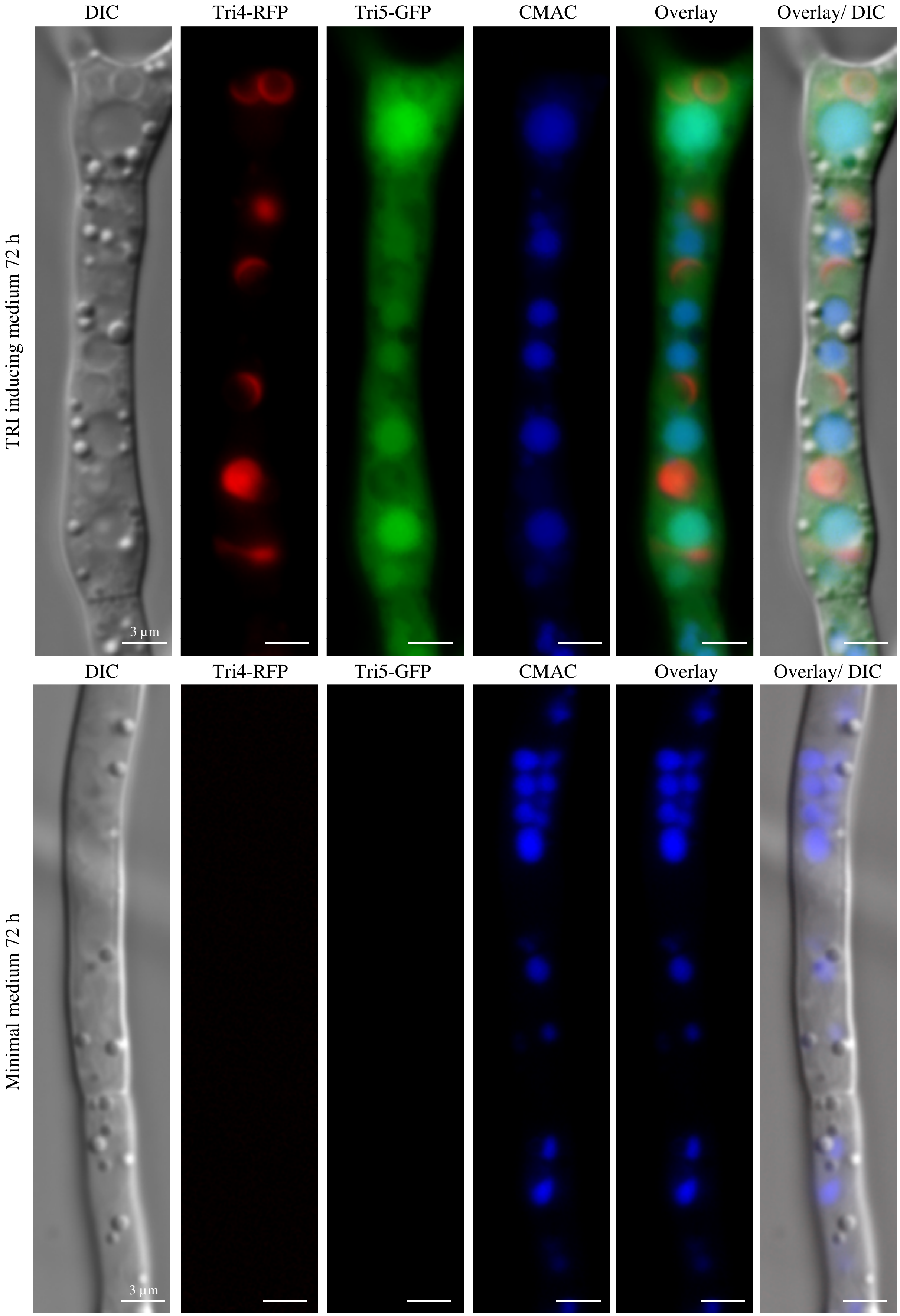
DIC
Tri4-RFP
Tri5-GFP
CMAC
Overlay
Overlay/ DIC
TRI inducing medium 72 h
3 µm
DIC
Tri4-RFP
Tri5-GFP
CMAC
Overlay
Overlay/ DIC
Minimal medium 72 h
3 µm

Supplementary Figure S5: Fluorescence microscopy of a Tri4-RFP/Tri5-GFP tagged strain grown in TIM (top) or MM (bottom) for 72 h stained with CMAC. TRI producing hyphae show Tri4-RFP fluorescence (red) of ER modifications, while Tri5-GFP fluorescence (green) is visible in the cytoplasm and in vacuoles (blue), as demonstrated by co-localization of GFP and the blue fluorescent dye CMAC, which accumulates in the vacuolar lumen. Hyphae of Tri4-RFP/Tri5-GFP grown in MM were stained with CMAC and imaged under similar conditions. Neither Tri4-RFP nor Tri5-GFP fluorescence are visible in MM, confirming that the expression of Tri4 and Tri5 is not induced in MM and that CMAC staining of vacuoles is functional under both growth conditions.

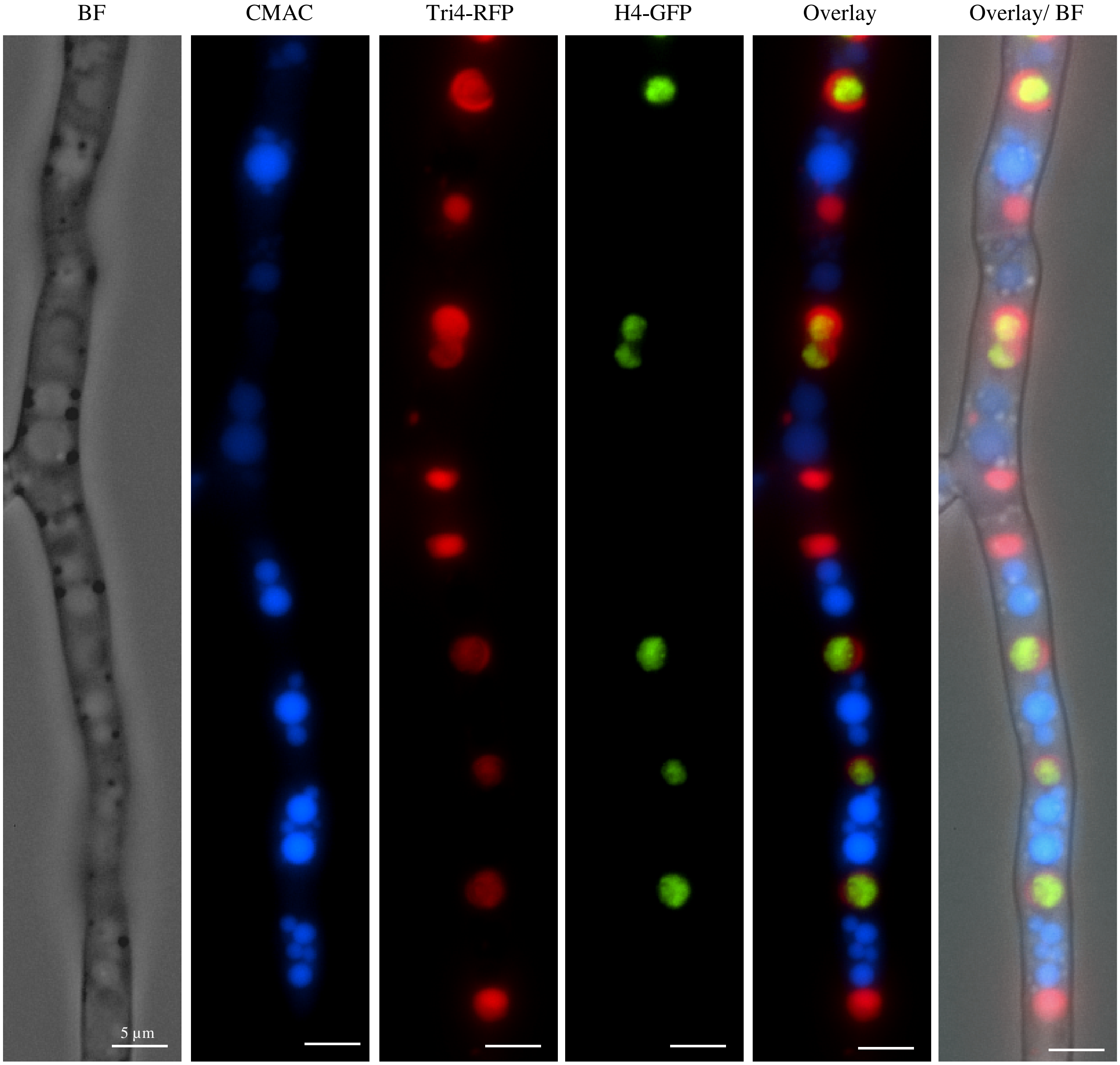

Supplementary Figure S6: Fluorescence microscopy of a H4-GFP/Tri4-RFP tagged strain grown in TIM for 48 h stained with the vacuolar lumen dye CMAC. In TRI producing hyphae Tri4-RFP fluorescence (red) of ER modifications is observed surrounding nuclei (green) and apart from nuclei, while vacuoles (blue), visualized by the blue fluorescent dye CMAC do not co-localize with ER modification or nuclei, demonstrating that neither the ER, nor nuclei are undergoing vacuolar degeneration under the conditions tested.

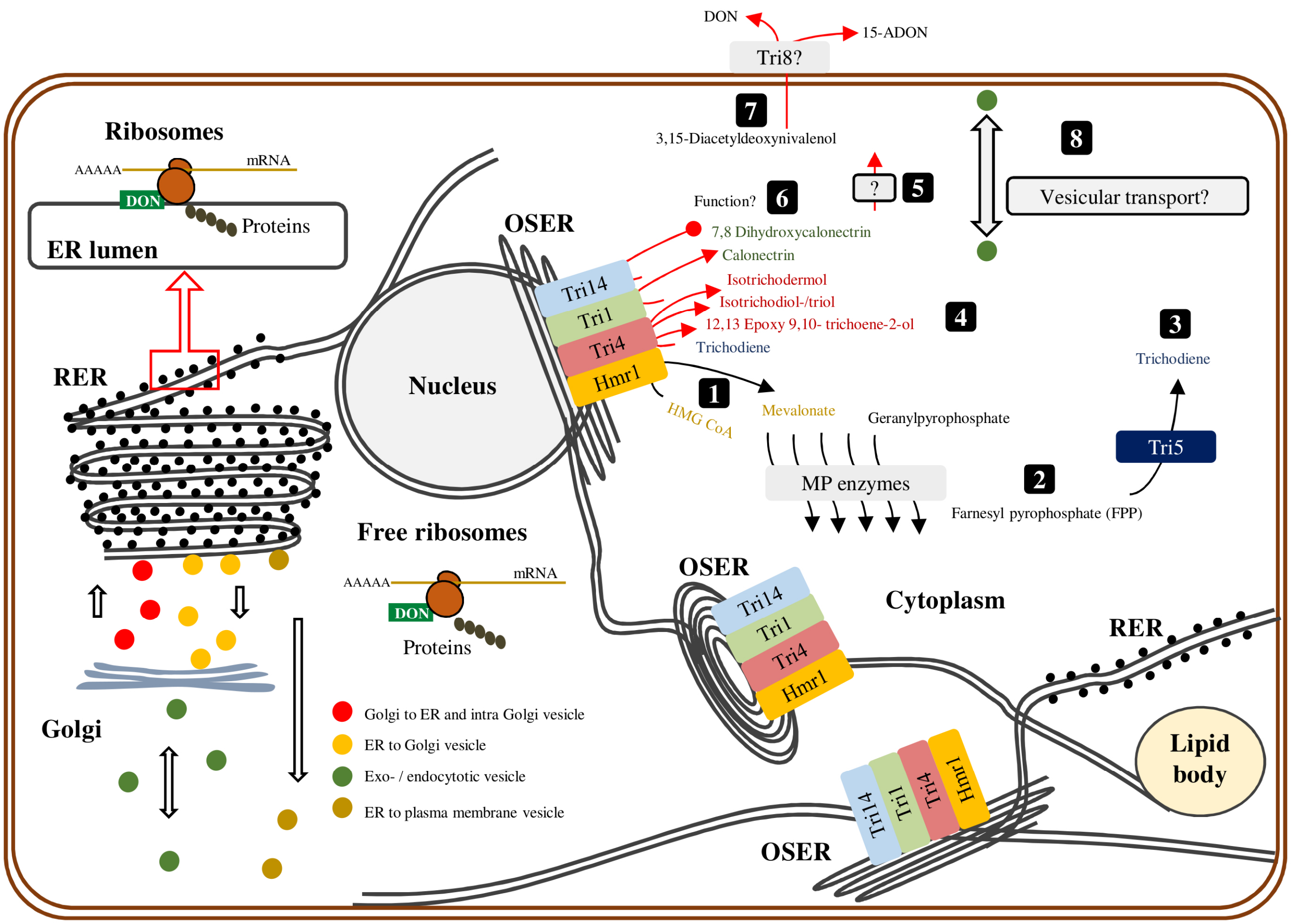

Supplementary Figure S7: Model of cellular synthesis of trichothecenes in *F. graminearum*. **1** The mevalonate pathway (MP) enzyme Hmr1 is localized at the ER and yields mevalonate and ultimately farnesyl pyrophosphate (FPP). **2** FPP is the substrate for the trichodiene synthase Tri5 producing trichodiene in the cytoplasm. **3** Trichodiene is oxygenated by OSER protein trichodiene oxygenase Tri4. **4** Tri4 generates the cytotoxic compound isotrichodermol. Isotrichodermol is modified by other Tri-enzymes (Tri101, Tri11 and Tri3) to calonectrin **5** OSER protein calonectrin oxygenase Tri1 generates 7,8 dihydroxycalonectrin, which is further converted to 3,15 diacetyldeoxynivalenol. **6** Tri14 is also localized at OSER, but its function is currently unknown. **7** Tri8 deacetylates 3,15 diacetyldeoxynivalenol and likely generates products 15-ADON and DON either inside or outside of the cell. **8** Toxic metabolites may be exported from OSER to outside of the cell by vesicles. TRI pathway enzymes within the cytoplasm of OSER may sequester toxic products and intermediates from their inhibitory targets, free ribosomes or rough ER. Lipid bodies are part of the ER network, but do not contain TRI enzymes, indicating that the ER might be organized in domains, harboring particular pathway enzymes.

Supplementary Movie legends:

Movie S1: 3D reconstruction of the ER of the *F. graminearum* wild type PH-1 grown in MM and stained with ER-Tracker. The ER was reconstructed based on a z-stack of 25 images of 3.48 x 12.99 µm in x- and y- dimension, 0.2 µm steps size between images, and a total thickness of 4.8 µm. Fluorescent structures turn around the x-axis of the z-stack.

Movie S2: 3D reconstruction of the ER of the *F. graminearum* wild type PH-1 grown in TIM and stained with ER-Tracker. The ER was reconstructed based on a z-stack of 19 images of 3.48 x 12.99 µm in x- and y- dimension, 0.2 µm steps size between images, and a total thickness of 3.6 µm. Fluorescent structures turn around the x-axis of the z-stack.

Movie S3: 3D reconstruction of Tri4-RFP, ER-Tracker, and BODIPY fluorescence in a hypha (see also Figure S1) grown in TIM. 3D reconstruction of a z-stack acquired by epi fluorescence microscopy show that ER-Tracker and BODIPY 493/503 co-localize within lipid bodies (green), while ER-Tracker (blue) and Tri4-RFP (red) co-localize both with crescent, spheres and ovoid structures. No co-localization between Tri4-RFP and lipid bodies is observed.

Supplementary Datasets:

Dataset S1: Proteomics data set and analysis used to generate heat map of enriched proteins in RFP plus and RFP minus fraction.

Dataset S2: Annotation of proteins enriched in RFP plus and RFP minus fraction.

Supplementary Methods

All primer sequences can be found in Supplementary Table S1. Proteomics data and annotation of proteins are available online in Supplementary Dataset S1 and S2 respectively. Key reagents, fungal strains, software, and their sources are provided in Supplementary Table S2.

Plant material and inoculation of wheat

Wheat plants *Triticum aestivum* cultivar Norm were grown from seeds in plastic pots filled with steamed topsoil and watered and fertilized as previously described [2], however, plants were grown in a plant growth chamber at 18 °C for 16 h (day) and 16°C for 8 h (night).

Inoculation of glumes and paleas of the wheat cultivar Norm with *F. graminearum* strains H4-GFP/Tri4-RFP and Tri1-GFP/Tri4-RFP was performed as described previously [3]. To study TRI producing infection structures of *F. graminearum*, paleas and glumes of wheat spikelets were isolated using a scalpel and placed on water agar (1.6% BD bacto dehydrated agar per liter) in 100×15 mm polysterene Petri dishes (Cat#25384-342, VWR, Radnor, PA, USA). Each assay consisted of 8 biological replicates of paleas or glumes in each of four petri dishes (n=32). All glumes and paleas were inoculated on the adaxial side with 5 µL, conidia suspension (containing 20 conidia /µL) in sterile water. Petri dishes were sealed with Parafilm and incubated in a plant growth chamber at 18°C for 16 h (day) and 16°C for 8 h (night) for 14 days. Infection structures were imaged by fluorescence microscopy, for paleas between 7 and 10 days post inoculation (dpi) and between 8 and 13 dpi for glumes.

Fungal growth conditions

For conidia production the fungus was grown in CMC culture and conidia were harvested as described previously [4]. Liquid minimal medium (MM) and trichothecene inducing medium (TIM) were adapted from a previous source [5] as described previously [6].

For conidia production, mycelia plaques of *F. graminearum* grown on potato dextrose agar (PDA) (12 g potato detrose 20 g BD bacto dehydrated agar per liter) for 3-5 days were inoculated in 50 mL liquid carboxymethylcellulose (CMC) medium [7] (30 g carboxmethylcellulose sodium salt, 2 g NH_4NO_3, 2 g KH_2PO_4, 1 g $MgSO_4$-$7H_2O$, 2 g yeast extract per liter). The fungus was grown for 6 d at 25°C under shaking at 150 rpm and conidia harvested as described previously [4]. Briefly, 25-50 mL CMC culture were filtered through Miracloth (Cat#475855 EMD Millipore, Billerica, MA, USA) into a 50 mL falcon tube and the filtrate centrifuged for 10 min at 3500g at 4°C. The pelleted conidia were washed with 25 mL sterile water by centrifugation as above and the pellet resuspended in 25 mL sterile water. Concentration of spores was determined using a hemacytometer (Cat#0267110, Fischer Scientific, Waltham, MA USA). Aliquots stored at -80°C were used to inoculate MM, TIM medium and paleas and glumes of wheat florets.

Liquid minimal medium (MM) and trichothecene inducing medium (TIM) was adapted from a previous source [5] as described previously [4] and contained per liter: 30 g of sucrose, 1 g of KH_2PO_4, 0.5 g of $MgSO_4$*$7H_2O$, 0.5 g of KCl, 10 mg of $FeSO_4$*$7H_2O$, 800 mg of putrescine dihydrochloride (5 mM final concentration), and 200 µL of trace element solution (5 g of citric acid, 5 g of

$ZnSO_4$*7H_2O, 0.25 g of $CuSO_4 5H_2O$, 50 mg of $MnSO_4$*H_2O, 50 mg of H_3BO_3, and 50 mg of $NaMoO_4$*2H_2O per 100 mL). Non TRI-inducing minimal medium (MM) was prepared as described for TRI-inducing medium, but instead of putrescine dihydrochloride, 0.848 g sodium nitrate ($NaNO_3$) (10 mM final concentration) was added as nitrogen source. The pH of both media was set to 4.5 with NaOH and sterile filtered through 0.22 µm filter (Cat#430767, Corning, Corning, NY, USA). 5 mL TIM or MM medium containing $1*10^4$ conidia in 14 mL plastic round bottom tubes (Cat#352059, Corning) were incubated with shaking at 25°C in the dark and grown hyphae were used for microscopy after 48 h if not noted otherwise.

Generation of GFP tagged strains

Reporter strains of *F. graminearum* wild type PH-1 (NRRL 31084) expressing GFP (Tab. 1, Supplementary Fig. S1) were generated using a fusion PCR and protoplast transformation as described earlier [6]. The dual tagged strain H4-GFP /Tri4-RFP, however, was generated by sexual crossing of strain Tri4-RFP and strain H4-GFP.

The tagging construct *pUCIDT-KAN:ER_GFP_HDEL* to generate strain GFP-HDEL was synthesized by IDT Integrated DNA Technologies (Coralville, IA, USA) in a *pUCIDT-KAN* vector. The tagging cassette contained the following sequences: *trpC* promoter from plasmid *pAL12-Lifeact* [8] (bases 2760 – 3117); ER targeting sequence from *Fusarium graminearum* FGSG_09471 (predicted BiP homolog, Genbank: XM_011329950) as predicted by SignalP-4.1, comprised of the start codon and following 96 base pairs (bases 222 – 320); GFP (without the start codon) from plasmid *pGFP::hph::loxP* (bases 1807 – 2523); HDEL ER retention signal and stop codon from FGSG_09471 (bases 2199 – 2213). Both the *trpC::GFP::HDEL* tagging cassette and neomycin resistance cassette were generated by PCR with Phusion Hot Start II High-Fidelity DNA Polymerase (see "Fusion PCR") and used to transform PH-1 protoplasts (see "Protoplast transformation") for ectopic insertion of both cassettes into the fungal genome (Supplementary Fig. S1a,b).

To generate strains GFP-HDEL, Sec22-GFP, Tri5-GFP/Tri4-RFP, Tri14-GFP/Tri4-RFP, and H4-GFP the *Neurospora* knock-in vector *pGFP::hph::loxP* [9] was used as a template to amplify a 2.35 kb *GFP::hph::loxP* fragment, while homologous flanks of 0.5-1 kb upstream and downstream of the stop codon of the gene to be tagged were amplified from genomic DNA of the wild type PH-1. The *GFP::hph::loxP* fragment was fused to homologous flanks using a fusion PCR method (see "Fusion PCR") and the resulting construct used to transform protoplasts (see "Protoplast transformation") and integrate the construct into the genome by homologous recombination (see Supplementary Fig. S1f-h). To generate stain H4-GFP, a split marker construct of the fusion construct was used for protoplast transformation (Supplementary Fig. S1m-p).

Fusion PCR

A fusion PCR method [10] was used to generate tagging constructs. The *Neurospora* knock-in vector *pGFP::hph::loxP* [9] was used as a template to amplify the 2.35 kb *GFP::hph::loxP* fragment, comprising of the 10x glycine linker, *GFP* ORF and the hygromycin B resistance gene (*hph*) driven by a *trpC* promoter as described earlier [6]. Homologous flanks of 0.5-1 kb upstream and downstream of the stop codon of the gene to be tagged were amplified from genomic DNA of the

wild type PH-1 using gene specific primers *LF1F* and *LF2R* (left flank) and *RF3F* and *RF4R* (right flank), while primers *GFPTagFwd* and *GFPTagRev* were used to amplify the *GFP::hph::loxP* fragment (see also Supplementary Tab. S1). The 5' ending of primers *LF2R* and *RF3F* contained 30 bases, reverse complement to *GFPTagFwd* or *GFPTagRev* primers, enabling fusion with the *GFP::hph::loxP* fragment by fusion PCR. PCR reactions were performed with Phusion Hot Start II High-Fidelity DNA Polymerase in 50 µL reactions containing: 10 µL 5x HF buffer, 1 µL Takara dNTPs (2.5 mM each dNTP), 1 µL each primer (10 µM), 35.5 µL water, 0.5 µL Phusion Taq and 1 µL genomic DNA (20-50 ng/µL) or vector *pGFP::hph::loxP* (1-2 ng/µL) as template. The following PCR conditions were applied: 1 cycle at 98°C for 1 min, 25 cycles at 98°C for 10 sec; annealing for 15 sec at 60°C for *GFP::hph::loxP* fragment (appropriate annealing temperature for LF and RF primers were used); extension was done at 72°C for 1 min and 15 sec to amplify the 2.35 kb *GFP::hph::loxP* fragment, but 15-23 sec for 0.5-1 kb flanks, 1 cycle at 72°C for 10 min. All three fragments were purified from agarose gels using Qiagen (Hilden, Germany) Gel Extraction Kit. For fusion PCR ~20 ng of the left and right fragment and ~15 ng *GFP::hph::loxP* fragment were pooled in a total volume of 6 µL. Fusion PCR reactions were performed in 50 µL total volume each as described above, but using 0.25 µL of 10 µM primers *LF1F* and *RF4R* (see Supplementary Tab. S1) and 32 µL water. The PCR program as described above was applied, but the appropriate annealing temperature for primers *LF1F* and *RF4R* was used respectively and the extension time at 72°C was increased according to the size of the fusion construct (23 sec/kb). Water instead of DNA template was used as negative control for each PCR reaction. The resulting fusion construct was gel purified from a band of expected fragment size using the same Qiagen Kit as above. Eluates were ethanol precipitated and resuspended in 10-20 µL buffer EB from Qiagen Gel Extraction Kit. 1-10 µg of the tagging construct was used for protoplast transformation (see below).

The *trpC::GFP::HDEL* cassette was amplified from plasmid *pUCIDT-KAN:ER_GFP_HDEL* and the neomycin resistance gene cassette from plasmid *pSM334* [11] by Fusion PCR using primer *HDELGFPF* and *HDELGFPR* (see Supplementary Fig. S1a and Supplementary Tab. S1) or primer *NEOF* and *NEOR* respectively (see Supplementary Fig. S1b and Supplementary Tab. S1) as described above, however annealing temperature appropriate for the respective primer pairs and extension time appropriate for respective product size was used. Both the *trpC::GFP::HDEL* cassette and neomycin resistance cassette were used to transform PH-1 protoplasts (see next section) and insert both cassettes ectopically into the fungal genome.

The strain H4-GFP was generated by using *10xGly-F* and *loxP-R* primers to amplify the 10x glycine linker, *GFP* and *hph* genes from *pGFP::hph::loxP* for fusion to up- and downstream flanks of FGSG_05491, the *F. graminearum hH4-2* gene (see Supplementary Tab. S1 for primer sequences). The 5' and 3' fusion products were amplified with primers *FgH4GlyF* and *hph-sm-r*, and *hph-sm-f* and *FgH4loxR2*, respectively to generate split-marker for transformation of protoplasts and integration of the construct into the genome by homologous recombination (Supplementary Fig. S1m-o). Genomic DNA extraction of mycelium from single vegetative spores of transformants was performed as previously described [12].

Protoplasts transformation

Protoplast preparation and fungal transformation were performed as described previously[6]. Briefly, protoplasts were isolated using 500 mg driselase and 200 mg lysing enzymes from *Trichoderma harzianum* in 20 mL 1.2 M KCl, incubated at 30°C with shaking at 80 rpm. Following protoplast formation, the suspension was filtered through two sheets of Miracloth (Cat#475855, EMD Millipore). The filtrate was centrifuged and the protoplast pellet washed three times with 1.2 M KCl. Final resuspension was to 10^7–10^8 protoplasts/mL in 93% 1.2 M STC (1.2 M sorbitol, 10 mM Tris-HCl (pH 8.0), 50 mM $CaCl_2$), 7% DMSO. Protoplasts were distributed into 200 mL aliquots which were stored at -80°C. Transformation of protoplasts was performed by adding 1–10 µg tagging construct to a 200 mL protoplast aliquot. Following 20 minutes incubation on ice, 1 mL 40% PEG 8000 in 1.2 M STC was added and the reaction incubated an additional 20 minutes at room temperature. The transformation reaction was transferred to 5 mL liquid TB3 (0.3% yeast extract, 0.3% casamino acids, 0.6 M sucrose) for regeneration, and cultures incubated for 16 h at 25°C with shaking at 150 rpm. Regenerated protoplasts were collected by centrifugation and resuspended in 1 mL 1.2 M STC. One third of the resuspension (approximately 350 µL) was added to 5 mL TB3 containing 0.7% low melting temperature agarose, and overlaid onto 7.5 mL solid TB3 (liquid TB3 with 0.7% low melting temperature agarose) containing 150 µg/ mL hygromycin B. Three selection plates were prepared for each transformation reaction. Plates were incubated for 16 hours at 25°C in the dark and then overlaid with 7.5 mL solid TB3 containing 250 µg/mL hygromycin B and incubated at 25°C in the dark until resistant colonies were visible (approximately 7 days). Resistant colonies were isolated onto V8 agar [13] (200 mL V8 juice, 2 g $CaCO_3$, 15 g BD bacto dehydrated agar per liter) containing 250 µg/mL hygromycin B and incubated for 7 days at 25°C. Conidia formed on V8 plates were spread on water agar for isolating single conidia of each putative transformant. Colonies grown from single conidia were used for further characterization and gDNA extraction. For selection with nourseothricin, regenerated protoplasts were overlaid onto solid TB3 containing 25 µg/mL nourseothricin, with the second TB3 overlay containing 50 µg/mL nourseothricin, and resistant colonies isolated onto PDA or V8 agar containing 50 µg/ml nourseothricin.

For selection of HDEL-GFP transformants with geneticin (neomycin resistance gene), regenerated protoplasts were overlaid onto solid TB3 containing 150 µg/mL geneticin, with the second TB3 overlay containing 250 µg/mL geneticin, and resistant colonies isolated onto V8 medium containing 150 µg/mL geneticin.

Verification of tagged transformants

Genomic DNA of transformants was extracted from freeze dried cultures, grown in liquid complete medium for 5 days at 25°C with shaking at 150 rpm, using the Omniprep DNA Kit as described previously [4]. Verification of integration of *GFP::HDEL* cassette and the neomycin resistance cassette into *F. graminearum* was done using primers amplifying the *promoter-GFP-HDEL* cassette (PCR1), the GFP coding region (PCR2), and primers amplifying the neomycin resistance gene cassette (PCR3) (see also Supplementary Tab. S1 and Supplementary Fig. S1a-e).

Verification of GFP tagging of genes *Sec22*, *Tri5*, and *Tri14* in genomic DNA of transformants was tested by PCR4 using primer pairs *TestExtF* and *TestExtR* (Supplementary Tab. S1 and

Supplementary Fig. S1f-l), which bind in the genomic locus downstream and upstream of the homologous flanks of the gene to be tagged (Supplementary Fig. S1f-h). Amplification with primer *TestExtF* and *TestExtR* will occur in both, the wild type and ectopic transformants, however in GFP tagged strains a PCR product 2.35 kb larger than in the wild type or ectopic strains will be amplified (Supplementary Fig. S1h-l). PCR reactions were performed with TaKaRa Ex Taq DNA Polymerase in 10 µL total volume, containing 1 µL gDNA (10-100 ng/µL) for transformants or wild type, 1 µL 10x ExTaq Buffer, 0.8 µL Takara dNTPs (2.5 mM each dNTP), 1 µL each primer (10 µM), 5.15 µL water and 0.05 µL of TaKaRa Ex Taq DNA Polymerase. PCR conditions were as follows: 1 cycle at 94 °C for 2 min; 25 cycles: 94 °C 1 min; annealing temperature appropriate for respective primers for 1 min; extension at 72 °C for 1 min/ 1 kb of expected fragment size; 1 cycle at 72 °C for 10 min. Water instead of DNA template was used as negative control for each PCR reaction.

Integration of split-marker fragments in genomic DNA of transformants H4-GFP resulted in insertion of a 2.3 kb construct with *GFP* and *hph* genes and detection of a 10.434 bp fragment when a *Bgl*II digest was probed with an *hph* fragment in two independent transformants by Southern blotting (Supplementary Fig. S1p), using Southern *hph* probe with the 5'-3' sequence AAAAAGCCTGAACTCACCGCGACGTCTGTCGAGAAGTTTCTGATCGAAAAGTTCGACAGCGTCTCCGACCTGATGCAGCTCTCGGAGGGCGAAGAATCTCGTGCTTTCAGCTTCGATGTAGGAGGGCGTGGATATGTCCTGCGGGTAAATAGCTGCGCCGATGGTTTCTACAAAGATCGTTATGTTTATCGGCACTTTGCATCGGCCGCGCTCCCGATTCCGGAAGTGCTTGACATTGGGGAGTTCAGCGAGAGCCTGACCTATTGCATCTCCCGCCGTGCACAGGGTGTCACGTTGCAAGACCTGCCTGAAACCGAACTGCCCGCTGTTCTCCAGCCGGTCGCGGAGGCCATGGATGCGATCGCTGCGGCCGATCTTAGCCAGACGAGCGGGTTCGGCCCATTCGGACCGCAAGGAATCGGTCAATACACTACATGGCGTGATTTCATATGCGCGATTGCTGATCCCCATGTGTATCACTGGCAAACTGTGATGGACGACACCGTCAGTGCGTCCGTCGCGCAGGCTCTCGATGAGCTGATGCTTTGGGCCGAGGACTGCCCCGAAGTCCGGCACCTCGTGCATGCGGATTTCGGCTCCAACAATGTCCTGACGGACAATGGCCGCATAACAGCGGTCATTGACTGGAGCGAGGCGA. Correct integration was also determined by PCR with primers *hph-sm-f* and *FgloxR1* (binding downstream of the 3' flank used for integration). Note that the *hH4-2* transcript extends into the 3' flank but the coding sequence ends at the *FgH4GlyR* primer, which excludes the stop codon.

Agarose gel electrophoresis of PCR products was performed using 0.8 or 1% SeaKem LE agarose in 0.5x TBE (TRIS-Borat-EDTA) buffer and 0.1% SYBRSafe in DMSO. Gel electrophoresis of PCR products and 1x loading dye (15% ficoll type 400, 0.25% bromphenol blue and 0.25% xylene cyanol FF in water) was performed at ~100 V for 45 min-1 h in 0.5x TBE.

Crossing procedure

In order to create a dual tagged strain H4-GFP /Tri4-RFP of *F. graminearum* sexual crossing was applied [14]. The single tagged strain H4-GFP #2 (Supplementary Fig. S1p), which contains the hygromycin resistance gene as selection maker and strain Tri4-RFP [6], which contains the nourseothricin resistance gene, were inoculated on carrot agar plates [15] (1.8% agar) for the mycelia to mix and form ascospores in perithecia. Seven carrot agar plates were inoculated with two mycelia plaques of H4-GFP and Tri4-RFP, obtained from colonies on PDA (agar containing either 150 µg/mL hygromycin B or 50 µg/mL nourseothricin respectively. Plaques were positioned alternate to each other as described previously [14]. Inoculated plates were incubated at 25°C and 12 h illumination per day. After 5 days, dense aerial mycelium on the plate was knocked down using a plastic spreader and 1 mL 2.5% Tween 60 solution per plate. Clumps of mycelia and agar plaques were discarded, plates were covered with a fresh lid, and incubated for 12 days under conditions

named. After 10-12 days perithecia were formed on the agar and ascospores were shot from perithecia to the lid of a Petri dish. Ascospores from the lid of seven identical plates were harvested using 1 mL sterile water. Ascospores were pooled and the spores screened for double resistant colonies using a sandwich agar method. Ascospores were spread on 15 mL PDA containing 150 µg/mL hygromycin B and colonies were grown for 2 days at 25°C. Colonies grown on plates were overlaid with 15 mL PDA containing 50 µg/mL nourseothricin. In total 1,050 ascospores were plated in three different concentrations (30, 60, and 120 ascospores/µl) on 5 replicate plates per concentration. 11 of the 38 double resistant colonies were picked from sandwich plates and grown on PDA under double selection (150 µg/mL hygromycin B and 50 µg/mL nourseothricin). Conidia of double resistant colonies were spread on 1.6% water agar under double selection as above and germinated overnight at 25 °C. Two germinated conidia of each colony were picked and grown at 25°C on PDA under double selection as above. After 3 days, flasks with liquid CMC medium were inoculated with mycelia plaques to obtain conidia. To screen for H4-GFP fluorescent nuclei and Tri4-RFP fluorescent ER proliferations in crossed strains, fluorescence microscopy of hyphae grown in TIM medium for 48h was performed.

Staining procedures

ER staining of hyphae was done with ER-Tracker Blue-White DPX (in the following named ER-Tracker) at a final concentration of 20 µM in equal volumes HBSS (Hank's Balanced Salt Solution) buffer pH 7.2 and two-fold concentrated MM or TRI inducing medium for 30 min at RT. Cut pipette tips were used to transfer 200 µL hyphae in liquid culture from MM or TIM to a 2 mL Eppendorf tube, containing 100 µL HBSS buffer pH 7.2 and 100 µL fresh minimal medium or TRI-inducing medium. Hyphae were incubated in fresh medium and the same HBSS buffer for 30 min at room temperature (RT) prior to ER-Tracker staining. To prepare the working stock 1 mM stock of ER-Tracker was diluted with the same buffer to a concentration of 40 µM and 500 µL dye was added to 500 µL hyphae in HBSS buffer. Staining was performed for 40 min at RT in the dark at a final concentration of 20 µM ER-Tracker. Stained hyphae were washed twice with equal volumes of HBSS buffer and 2-fold concentrated MM or TIM respectively. 100 µL stained and washed hyphae were mounted on glass slides using wide mouth pipette tips. Counterstaining of ER-Tracker and BODIPY 493/503 was done by adding BODIPY at a final concentration of 0.05 µM to ER-Tracker stained hyphae and incubation for 15 min at RT.

Staining of the lumen of vacuoles in hyphae was performed with CellTracker Blue dye CMAC (7-amino-4-chloromethylcoumarin) (named CMAC in the following) at a final concentration of 100 µM CMAC and 1% DMSO as described previously [4]. 100 µL hyphae grown in MM or TIM liquid culture were transferred into a 2.5 mL tube containing 100 µL of 200 µM CMAC (2% DMSO) diluted with PBS pH 7.2 and stained for 30 min at RT. Hyphae were washed with 2 mL of equal volumes PBS buffer pH 7.2 and 2-fold concentrated minimal medium or TRI inducing medium respectively.

To visualize fungal cell walls of infection structures of the fungus *in planta*, 0.1% Calcofluor White stock solution was diluted with PBS buffer pH 7.2 to a working concentration of 0.001% Calcofluor White. 1-3 mm^2 sections of wheat glumes and paleas showing infection structures were placed in Calcofluor White working stock for 15 min and washed twice in fresh PBS buffer prior to microscopy.

Epifluorescence microscopy

Cultures used for microscopy were grown for 48 h in MM or TRI inducing medium. All samples were mounted on glass slides (Cat#3010, Gold Seal Plain Microslides, Thermo Fischer Scientific) and sealed with cover glasses (Cat#2980-223, Coning). Epifluorescence microscopy was performed using Nikon ECLIPSE90i microscope (Nikon, Melville, USA), equipped with Nikon mercury lamp (Cat#MBF74650), Nikon 100x objective, and camera DS-Qi1Mc-U2 (12 bit 1280x1024 pixel). The following filter sets were used: Filter set Nikon C-FL GFP HC HISN Zero Shift (λ_{ex} 450-490/ λ_{em} 500-550) for green fluorescence, filter set Nikon DAPI: C-FL DAPI HC HISN Zero Shift (λ_{ex}325-375/ λ_{em} 435-485) for blue fluorescence, and Chroma filter set HcRed1 (λ_{ex} 550-600/ λ_{em} 615-665) (Chroma Technology Corporation, Bellows Falls, USA) for RFP fluorescence. All images were acquired using multichannel z-stacks of hyphae. Microscopy raw images were cropped and processed similarly in MM and TRI and fluorescence channels. 3D reconstruction shown in Movie 3 was generated from a z-stack using the shaded surface rendering function of Nikon NIS elements AR software 4.30.01.

Cultures imaged by bright field (BF) or differential interference contrast (DIC) and fluorescence microscopy (FM) were grown for 48 h in MM or TIM. FM was performed using Nikon ECLIPSE90i microscope (Nikon, Melville, USA). A z-stack image is shown in Figure 2a-d, 3a and b (bottom images), 5a (left), and Supplementary Figure S3a. Maximum intensity projections (MIPs) of z-stack images are shown in Figure 3b (top image), 4b and c, 5a (right), 5b-d, Supplementary Figure S2, S5, and S6. Bright field (BF) or difference interference contrast (DIC), and overlay images with BF or DIC, show a z-stack image from the center of the respective cell, except for the overlay with BF in Figure 3b (top image) and Supplementary Figure S6 which are MIPs.

Super resolution microscopy

Super resolution microscopy images of the ER visualized by ER-Tracker were acquired using a Nikon N-SIM system at the University of Minnesota imaging center (UIC) in Minneapolis, Minnesota, USA. The Nikon N-SIM system consisted of an inverted Nikon Ti-E microscope, equipped with a Nikon structured illumination system and an Andor DU-897 X-8444 camera. The microscope was used at the University of Minnesota Imaging Center with technical support by Guillermo Marques. Laser light of 405 nm on power settings from 700-900% was used to excite ER-Tracker and its fluorescence emission was detected at 430-490 nm using Apo TIRF 100x Oil DIC N2 (NA: 1.49 refractive index: 1.515) objective. Z-stacks of 45x45 µm in x/y and 6-8 µm total thickness were acquired in 0.2 µm steps using 3D SIM acquisition mode. Similar fluorescence intensities of all z-stacks was controlled by gray values of pixels in “look up table” (LUT) graphs. Z-stack images were acquired using exposure times of 100-400 msec depending on signal intensity. The following settings were used to reconstruct super resolution images: Low contrast off; contrast 0.50; apodization 0.50; Width3DFilter 0.10; optimize on. The Nikon NIS Elements AR software 4.20.02 was used for image acquisition and SIM reconstruction. Hyphae grown in MM or TIM were mounted on glass slides (Cat#3010 Gold Seal Plain Microslides) and covered with high precision 18x18 mm glass coverslips with a thickness of 170 nm ± 5 µm (Cat#0107032, Marienfeld Superior, Lauda Königshofen, Germany). Figure 3e row 1 shows the SIM reconstruction of one image of a z-stack from the center of a cell grown in MM and TIM. The blue

fluorescence of ER-Tracker was displayed in a pale yellow color. A representative cell in MM and TIM was similarly cropped from original image files and 3D reconstructed using the shaded surface rendering function of the Nikon NIS elements AR software 4.30.01.

Transmission electron microscopy (TEM)

For TEM cultures were grown in liquid minimal medium (MM) and TRI inducing medium (TIM) for 65 h. 3-4 h before high pressure freezing (HPF) the cultures were washed twice with sterile water and transferred into MM and TIM containing 1% sucrose. The mycelium was filtered through Miracloth (Cat# 475855, EMD Millipore) and placed on Whatman filter paper (Cat#WHA1001070, Sigma-Aldrich, St. Louis, MO, USA)) and a dense ball of mycelium was formed using a sterile plastic stick and the mycelium transferred with a syringe needle to brass hats of 200 µl volume. Four repetitive samples from grown in MM and TRI medium were high pressure frozen using Balzers HPM 010 according to the protocol described previously [16]. Brass hats containing HPF samples were stored in liquid nitrogen (N_2) until freeze substitution (FS) was performed. Samples were transferred into vials, which were cooled in liquid N_2 containing and contained freeze substitution (FS) solution (1% glutaraldehyde, 2% osmium tetroxide, 0.1% uranyl acetate in methanol, 1.9% methanol and 5% water in acetone). The FS solution was produced as "Müller-Reichert and Antony Freeze Substitution Cocktail" [17], however 5% water was added to improve visibility of ER membranes [18, 19]. Briefly, 10 mL fixative in acetone was produced by solving 0.2 g osmium tetroxide in 8 mL acetone and adding 1 mL 10% glutaraldehyde in acetone, 0.2 mL 5% uranyl acetate in methanol [17] and 0.5 mL water. 1 mL FS solution was pipetted into each of 8 cryovials and cooled with liquid N_2. Brass hats containing samples were transferred quickly from vials in liquid N_2 into cryovials containing FS medium. Vials were closed quickly and transferred into in Leica AFS2 chamber (Leica Microsystems, Buffalo Grove, IL, USA) precooled at -90° C and hold at the temperature for 28 h. The samples were gradually warmed up to 20°C by the following steps: 1.) warm up by 3°C/h to -60°C and hold for 2 h, 2.) warm up by 8°C/h [20] to -20°C and hold for 12 h [17], 3.) warm up to 4°C at a rate of 5°C/h and hold at for 4 h [16], 4.) warm up to 20°C at a rate of 10°C/h [17]. Samples were washed with acetone every 15 min over a 1 h period and infiltrated with resin at room temperature as described earlier [16]. The epoxy resin Poly/Bed 812 (Luft formulations) Embedding Kit was used and polymerized for 48 h at 60°C or 24 h. Ultramicrotome sections of ~70 nm thickness were collected on TEM grids and poststained with Reynold's lead citrate lead citrate [21] (Reynolds, 1963) for 2 min. Transmission electron micrographs were done with TEM FEI Tecnai T12 operating at 60 kV.

Proteomics of ER membranes

ER membranes formed in a Tri4-RFP tagged strain after 48 h in TIM were enriched from cellular lysates of protoplasts using Fluorescence-Activated Cell Sorting (FACS) (see section below). Particles in the lysate were assessed for RFP fluorescence intensity and size and sorted into "RFP plus" and "RFP minus" fractions (see also Supplementary Figure S3). Extracted and digested peptides of both fractions from three biological replicates were identified by mass spectrometry, MS/MS analysis and comparative proteomics (see sections below and Supplementary Dataset S1, S2)

Fluorescence-Activated Cell Sorting (FACS)

This protocol was modified from [22]. The mycelium of Tri4-RFP strain grown in TRI inducing medium for 48 h was harvested by filtering with 2-fold Miracloth (Cat#475855, EMD Millipore) and washed first with 50 mL sterile Millipore water, then twice with 35 mL 0.7 M NaCl and 0.01 M NaH_2PO_4 at pH 5.8. Mycelia was collected from the Miracloth and added to 20 mL enzyme solution (100 mg Kitalase, 0.7 M NaCl and 0.01 M NaH_2PO_4, pH 5.8 filter sterilized through 0.22 µm filter (Cat#430767, Corning). The mycelia in enzyme solution was incubated at 30°C with shaking at 50 rpm for ~45 min until protoplasts formed. Digested mycelia was filtered through a funnel with 2 fold sterile Miracloth into a 50 mL falcon tube, the funnel with Miracloth was transferred to a sterile 250 mL Erlenmeyer flask and the Miracloth washed twice with 25 mL 0.7 M NaCl and 0.01 M NaH_2PO_4 pH 5.8. Protoplasts in solution were centrifuged at 1300g (2,500 rpm in SH-3000 rotor) for 5 min and the pellet resuspended in 10 mL 1.2 M sorbitol, 10 mM Tris pH 8.0. The centrifuge and resuspension step was repeated, and then the protoplasts were counted. Protoplasts were centrifuged again as above, but for 10 min, and the protoplast pellet resuspended in 1.2 M sorbitol-Tris pH 8.0 to the desired concentration. Lysis of protoplasts was modified from [23]. Protoplasts were lysed by adding 5 volumes 0.6 M sorbitol 10 mM Tris pH 8.0 to 1 volume protoplasts for a final concentration of 0.7 M sorbitol. Samples were stored at -80°C until peptide extraction and digestion was performed. The flow cytometry data analysis and FACS population enrichment were obtained using a BD Influx Fluorescence-Activated Cell Sorter (BD Biosciences, San Jose, CA, USA). Optimization and calibration of the fluorescence-activated cell sorting was performed before each analysis using 3 µm Ultra Rainbow Fluorescent Particles (Cat# URFP-30-2 Spherotech, Lake Forest, IL, USA). Lysed *Fusarium graminearum* protoplasts were diluted with 100 mM ammonium bicarbonate (Cat# A6141, Sigma-Aldrich) and passed through a 35 mm strainer cap (Cat# 352235 Corning) to remove large aggregates. Ammonium bicarbonate allowed improved recovery in the preparation of proteomic samples. Sheath fluid consisted of 100 mM ammonium bicarbonate. Using a 100-µm nozzle, red fluorescent protein (RFP) was excited using the 561 nm excitation from a Cobolt Jive 75 (Cat# 0561-04-01-0075-1001 Cobolt, Solna, Sweden) at 75 mW. Forward scatter and RFP intensity were used to identify and gate the RFP containing TAO population, and the non-fluorescent control population of the same size. A minimum $1E^6$ events were enriched for each population. Enriched samples are then pelleted at 2,000g. The supernatant is aspirated and the pellet fraction is stored at -80°C.

Peptide extraction and digestion

A SDS-lysis buffer (4% SDS (w/v), 100 mM Tris/HCl pH 7.6, 100 mM DTT (Dithiothreitol)) was added to each sample and incubated at 95°C for 5 min to completely reduce and denature the protein. The samples were cooled at 4°C for 30 min and centrifuged at 15,000g for 10 mins to pellet any remaining debris. Filter Aided Sample Preparation (FASP) [24] kits were used for protein digestion (Cat#44250 Expedeon, San Diego, CA, USA) according to the manufacturer's instructions. Briefly, 400 µL of 8M urea (all reagents included in the kit) was added to each 500 µL 30K molecular weight cut off (MWCO) FASP spin column and up to 100 µL of the sample in SDS buffer was added, centrifuged at 14,000g for 30 mins to bring the sample all the way to the dead volume. The waste was removed from the bottom of the tube and another 400 µL of 8M urea was added to the column and centrifuged again at 1,4000g for 30 min and repeated once more. 400 µL of 50mM ammonium bicarbonate (provided) was added to each column and centrifuged for 30

min, was done twice. The column was placed into a new fresh, clean and labeled collection tube. Digestion solution was made by dissolving 4 µg trypsin in 75 µL 50 mM ammonium bicarbonate solution and added to the sample. Each sample was incubated for 3 h at 37°C with 800 rpm shaking on a thermomixer with a thermotop (Eppendorf, Hamburg, Germany) to reduce condensation into the cap. The resultant peptides were then centrifuged through the filter and into the collection tube at 1,4000g for 15 min. The peptides were snap frozen in liquid N_2 and the column placed back into the collection tube and digested again overnight, the following day the peptides were added to the 3 h peptides and concentrated to ~20 µL using a SpeedVac.

Mass spectrometry and MS/MS data search

All data were collected on hybrid Velos linear ion trap coupled Orbitrap mass spectrometers (Thermo Electron, Waltham, MA, USA) coupled to Waters NanoAcquity or Next-Gen 3 high performance liquid chromatography systems (Cat#176016000 Waters Corporation, Milford, MA, USA) through 75 µm x 70 cm columns packed with Phenomenex Jupiter C-18 derivatized 3 µm silica beads (Cat#04A-4053 Phenomenex, Torrance, CA, USA). Samples were loaded onto columns with 0.05% formic acid in water and eluted with 0.05% formic acid in Acetonitrile over 100 minutes. Ten data-dependent MS/MS scans were recorded for each survey MS scan using normalized collision energy of 35, isolation width of 2.00, and rolling exclusion window of +1.55/-0.55. The lasting 60 seconds before previously fragmented signals are eligible for re-analysis. The MS/MS spectra from all LC-MS/MS datasets were converted to ASCII text (.dta format) using DeconMSn [25] which more precisely assigns the charge and parent mass values to an MS/MS spectrum. The data files were then interrogated via target-decoy approach [25] using MSGFPlus [26] with a +/- 50 ppm parent mass tolerance, no specific digestion enzyme settings, and a variable posttranslational modification of oxidized Methionine. All MS/MS search results for each dataset were collated into tab separated ASCII text files listing the best scoring identification for each spectrum.

Protein identification and data analysis

As a reference for identification, the annotated chromosomal protein sequences for *F. graminearum* were obtained from the Broad Institute (13,321 entries, http://www.broadinstitute.org/annotation/genome/fusarium_group/Downloads.html) and combined with mitochondrial sequences from the same organism obtained from Genbank (50 entries, http://www.ncbi.nlm.nih.gov/nuccore/DQ364632.1). Further, the quadricolor synthetic red fluorescent protein sequence was obtained from (Genbank: ACD03281.1), and lastly common contaminant proteins (trypsin, keratins, etc.) were added to the final search file. All sequences were loaded into a SQL Server relational database structure to allow on-the-fly FASTA format file creation for MS/MS peptide-to-spectrum matching (PSM) analysis. Collated search results were combined into a single raw file. These results were imported into a Microsoft SQL Server database. Using decoy identifications (exactly reversed peptide sequences denoted as "XXX_" in their protein references), we assessed the peptide-to-spectrum-match (PSM) false discovery levels as (2x#ReverseIDs)/(#filterPassingIDs). This assumes the count of reverse PSMs only reports half of all likely to be false filter passing identifications) and results were filtered to approximately 1% false discovery. Filter passing results were reported in an Excel file. Using the protein references as a grouping term, unique peptides belonging to each protein were counted, as were all PSMs

belonging to all peptides for that protein (i.e. a protein level observation count value). PSM observation counts reported for each sample that was analyzed. Cross-tabulation tables were created to enumerate protein level PSM observations for each sample, allowing low-precision quantitative comparisons to be made. Identified proteins enriched in RFP plus and RFP minus fractions (Supplementary Dataset S1) were analyzed to further determine annotation and predicted function (Supplementary Dataset S2). The JGI *Fusarium graminearum* v 1.0 database (http://genome.jgi.doe.gov/Fusgr1/Fusgr1.home.html) and the KEGG Genome *Fusarium graminearum* database (http://www.genome.jp/kegg-bin/show_organism?org=fgr) were queried to find updated annotations. To identify homologs, BLASTP searches using the protein sequences from JGI were performed using both the *Saccharomyces cerevisiae* genome database (http://www.yeastgenome.org/blast-sgd, YeastORF.pep) and NCBI human database (http://blast.ncbi.nlm.nih.gov/Blast.cgi, Homo sapiens taxid:9606). Homologs were annotated if E-value was less than 5E-50. Proteins sequences were examined to determine if they contained a C- terminus KDEL or HDEL ER retention signal. Proteins also were examined to determine if they contained predicted ER targeting signal peptides by using SignalP-4.1 (http://www.cbs.dtu.dk/services/SignalP/). Proteomics heat map was generated with the heatmap.2 function in the R software package Gplots, using R version 3.2.3.

Supplementary Table S1: Primers used for GFP tagging of *F. graminearum* proteins and primers used to confirm GFP tagging of transformants (see also Supplementary Figure S1).

Amplicons	Primer name	Sequence (5' to 3')
TrpC::GFP::HDEL cassette	*HDELGFPF*	CAACTGGATATTGAAGGAAGCA
	HDELGFPR	TTACAACTCGTCGTGCTT
Neomycin resistance gene cassette	*NEOF*	GAATTGGGTACTCAAATTGGTTCCC
	NEOR	ATATGCATAGTACCGAGAAACTAGGC
GFP::hph::loxP cassette	*GFPTagFwd*	GGCGGAGGCGGCGGAGGCGGAGGCGGAGGC
	GFPTagRev	GACCATGATTACGCCAAGCTATTTAGGTGA
Upstream (LF) and downstream (RF) regions flanking the *Sec22* stop codon	*Sec22GFPTagLF1F*	TCTTTATCGTAGCGCAAGCA
	Sec22GFPTagLF2R	GCCTCCGCCTCCGCCTCCGCCGCCTCCGCCAAA GAATCGCCAATACAGGAA
	Sec22GFPTagRF3F	TCACCTAAATAGCTTGGCGTAATCATGGTCGA AATGCAAGCGAGCTAGTTCTGTT
	Sec22GFPTagRF4R	ATCGCGACATTGCCTTTGT
Upstream (LF) and downstream (RF) regions flanking the *Tri5* stop codon	*Tri5GFPTagLF1F*	GTGGGCACTTGTCAACGAGCACT
	Tri5GFPTagLF2R	GCCTCCGCCTCCGCCTCCGCCGCCTCCGCC CTCCACTAGCTCAATTGAACTTA
	Tri5GFPTagRF3F	TCACCTAAATAGCTTGGCGTAATCATGGTCCCG AAGGCGAGTTTGGAAGTATG
	Tri5GFPTagRF4R	GAAGTTGGGGGAAAGACGGGAAG
Upstream (LF) and downstream (RF) regions flanking the *Tri14* stop codon	*Tri14GFPTagLF1F*	GTGATGGTTTCAACGTGTCG
	Tri14GFPTagLF2R	GCCTCCGCCTCCGCCTCCGCCGCCTCCGCCGAT CTCGTCGCGGACCTC
	Tri14GFPTagRF3F	TCACCTAAATAGCTTGGCGTAATCATGGTCTAG GCTCAACTGTAAGATGATGG
	Tri14GFPTagRF4R	ATGCATGACGTCCATCAAAA
Primers used to test GFP tagging in transformants of *GFP-HDEL*, *Sec22-GFP Tri5-GFP* and *Tri14*-GFP	*HDELGFPcodF*	TGCTCAGGTAGTGGTTGTCG
	HDELGFPcodR	ACGTAAACGGCCACAAGTTC
	Sec22GFPTagTestExtF	AACGAACCACATTGAGAACC
	Sec22GFPTagTestExtR	TGGGCAATGGATAAAACAGT
	Tri5GFPTagTestExtF	AGTGCATGGCGGATCTATCTA
	Tri5GFPTagTestExtR	TTTGGGTTGATGCATTGCAG
	Tri14GFPTagTestExtF	GGATTACCCAGTGGGTTGAGAAA
	Tri14GFPTagTestExtR	TTCGATGACGGTCCGTTTATATAC
Upstream (LF) and downstream (RF) regions flanking the *H4* stop codon and primers* used to generate split marker	*FgH4GlyF**	GTTTGGAGGTGGCATATATCG
	FgH4GlyR	CCTCCGCCTCCGCCTCCGCCGCCTCCGCCACCA CCGAAACCGTAGAGGGTAC
	FgH4loxF	TGCTATACGAAGTTATGGATCCGAGCTCGAGC TATCTGCACACCGAATGG
	*FgH4loxR2**	TTGGATGCACTACGCGACACC
	*hph sm-r**	TCGCCTCGCTCCAGTCAATGACC
GFP::hph::loxP cassette	*10XGly-F*	GGCGGAGGCGGCGGAGGCGGAGGCGGAGG
	loxP-R	CGAGCTCGGATCCATAACTTCGTATAGCA
Primers used to test GFP tagging in *H4-GFP* transformants	*FgH4loxR1*	TATGAACAAATCTACCGGACT
	*hph sm-f**	AAAAAGCCTGAACTCACCGCGACG

Supplementary Table S2: Key reagents, fungal strains, and software used in this study.

REAGENT	SOURCE	IDENTIFIER
**Chemicals, dyes, proteins**		
Ammonium bicarbonate	Sigma-Aldrich	Cat#A6141
Bactopeptone	Thermo Fisher Scientific	Cat#DF0118170
BBL yeast extract	BD	Cat#211929
BD bacto dehydrated agar	Thermo Fisher Scientific	Cat#DF0140010
BODIPY 493/503	Thermo Fischer Scientific	Cat#D3922
Calcofluor White	Sigma-Aldrich	Cat#1809
Casamino acids	GSS	Cat#BP1424100
CellTracker Blue CMAC	Thermo Fischer Scientific	Cat#C2110
CMC	Sigma-Aldrich	Cat#5678
Driselase	Sigma-Aldrich	Cat#D9515-5G
ER-Tracker™ Blue-White DPX	Thermo Fischer Scientific	Cat#E-12353
Geneticin	Invitrogen/ Gibco	Cat#10131-035
HBSS buffer pH 7.2	Thermo Fisher Scientific	Cat#14025
Hygromycin B	Sigma-Aldrich	Cat#10843555001
Kitalase	Wako Chemicals USA	Cat#114-00373
Low melting temperature agarose	Lonza	Cat#50080
Lysing enzymes	Sigma-Aldrich	Cat#L1412-10G
Nourseothricin	United States Biological	Cat#N5375-74
PBS buffer pH 7.2	Thermo Fisher Scientific	Cat#20012043
PEG 8000	Sigma-Aldrich	Cat#25322-68-3
Potato dextrose broth	Thermo Fisher Scientific	DF0549179
Putrescine dihydrochloride	Sigma-Aldrich	Cat#P5780-25G
SeaKem LE agarose	Lonza	Cat#5004
Sodium nitrate	Thermo Fisher Scientific	Cat#BP360-500
V8 juice Original	Campbells Soup Company	https://www.campbells.com/v8/vegetable-juice
**Critical Commercial Assays**		
Omniprep DNA kit	G Biosciences	Cat#786-136
Qiagen Gel Extraction Kit	Qiagen	Cat#28706
Phusion Hot Start II High-Fidelity DNA Polymerase	Thermo Fisher Scientific	Cat#F-549L
TaKaRa Ex Taq DNA Polymerase	Clontech	Cat#RR001A
TaKaRa dNTPs	Clontech	Cat#4030
100 bp DNA ladder	NEB	Cat#N3231L
1 kb DNA ladder	NEB	Cat#N3232L
SYBRSafe in DMSO	Invitrogen	Cat#S33102
Poly/Bed 812 (Luft formulations) Embedding Kit	Polysciences Inc.	Cat#08792-1
Filter Aided Sample Preparation (FASP) kit	Expedeon	Cat#44250

**Table S2 continued**		
**Organisms/Strains**		
F. graminearum wild type PH-1 (NRRL 31084)		Fungal Genetics Stock Center Kansas State, USA
Hmr1-GFP strain	Lab H. Corby Kistler	Menke et al., 2013[6]
GFP-HDEL	Lab H. Corby Kistler	This paper
Sec22-GFP	Lab H. Corby Kistler	This paper
Tri4-RFP	Lab H. Corby Kistler	Menke et al., 2013 [6]
Tri1-GFP/Tri4-RFP	Lab H. Corby Kistler	Menke et al., 2013[6]
Tri5-GFP/Tri4-RFP	Lab H. Corby Kistler	This paper
Tri14-GFP/Tri4-RFP	Lab H. Corby Kistler	This paper
H4-GFP	Lab Michael Freitag	This paper
H4-GFP/Tri4-RFP	Lab H. Corby Kistler	This paper
**Recombinant DNA**		
pGFP::hph::loxP	GenBank ID#FJ457011.1	Honda and Selker, 2009 [9]
pUCIDT-KAN	IDT Integrated DNA Technologies	Cat#
pUCIDT-KAN:ER_GFP_HDEL	Lab H. Corby Kistler	This paper
pSM334	Lab Seogchan Kang	Hou et al., 2002 [11]
pAL12-Lifeact	Lab Nick D. Read	Lichius et al., 2010 [8]
**Software and Algorithms**		
SignalP-4.1	http://www.cbs.dtu.dk/services/SignalP/	Petersen et al., 2011
NIS Elements AR Version 4.20.02 and 4.30.01	Nikon Instruments Inc.	https://www.nikoninstruments.com/Products/Software/NIS-Elements-Advanced-Research
R version 3.2.3	https://www.R-project.org/	R Core Team 2015
Heatmap.2 function in Gplots R package	https://CRAN.R-project.org/package=gplots	Warnes et al., 2015
MSGFPlus	http://proteomics.ucsd.edu/Software/MSGFPlus/#Downloads	Kim and Pevzner, 2014 [26]

References

1. Kanehisa, M. & Goto, S. KEGG: kyoto encyclopedia of genes and genomes. *Nucleic Acids Res.* **28**, 27-30 (2000).
2. Goswami, R.S. & Kistler, H.C. Pathogenicity and *in planta* mycotoxin accumulation among members of the *Fusarium graminearum* species complex on wheat and rice. *Phytopathology* **95**, 1397-1404 (2005).
3. Boenisch, M.J. & Schäfer, W. *Fusarium graminearum* forms mycotoxin producing infection structures on wheat. *BMC Plant Biol.* **11**, 110 (2011).
4. Menke, J., Dong, Y.H. & Kistler, H.C. *Fusarium graminearum* Tri12p influences virulence to wheat and trichothecene accumulation. *Mol. Plant-Microbe Interact.* **25**, 1408-1418 (2012).
5. Gardiner, D.M., Kazan, K. & Manners, J.M. Nutrient profiling reveals potent inducers of trichothecene biosynthesis in *Fusarium graminearum. Fungal Genet. Biol.* **46**, 604-613 (2009).
6. Menke, J., Weber, J., Broz, K. & Kistler, H.C. Cellular development associated with induced mycotoxin synthesis in the filamentous fungus *Fusarium graminearum. PLoS One* **8**, 12 (2013).
7. Cappellini, R. & Peterson, J. Macroconidium formation in submerged cultures by a non-sporulating strain of *Gibberella zeae*. *Mycologia* **57**, 962-966 (1965).
8. Lichius, A. & Read, N.D. A versatile set of Lifeact-RFP expression plasmids for live-cell imaging of F-actin in filamentous fungi. *Fungal Genet Rep* **57**, 8-14 (2010).
9. Honda, S. & Selker, E.U. Tools for fungal proteomics: multifunctional neurospora vectors for gene replacement, protein expression and protein purification. *Genetics* **182**, 11-23 (2009).
10. Szewczyk, E. *et al.* Fusion PCR and gene targeting in Aspergillus nidulans. *Nat. Protoc.* **1**, 3111-3120 (2006).
11. Hou, Z. *et al.* A mitogen-activated protein kinase gene (MGV1) in *Fusarium graminearum* is required for female fertility, heterokaryon formation, and plant infection. *Mol. Plant-Microbe Interact.* **15**, 1119-1127 (2002).
12. Pomraning, K.R., Smith, K.M. & Freitag, M. Genome-wide high throughput analysis of DNA methylation in eukaryotes. *Methods* **47**, 142-150 (2009).
13. Simmons, E.G. Alternaria taxonomy: Current status, viewpoint, challenge., in *Alternaria: Biology, plant disease and metabolites,*. (ed. J. Chelkowski. A. Visconti) 1-35 (Elsevier Science Ltd., Amsterdam; 1992).
14. Bowden, R.L. & Leslie, J.F. Sexual recombination in *Gibberella zeae*. *Phytopathology* **89**, 182-188 (1999).
15. Klittich, C. & Leslie, J.F. Nitrate reduction mutants of *Fusarium moniliforme* (*Gibberella fujikuroi*). *Genetics* **118**, 417-423 (1988).
16. Murray, S. High pressure freezing and freeze substitution of Schizosaccharomyces pombe and Saccharomyces cerevisiae for TEM. *Methods Cell Biol.* **88**, 3-17 (2008).
17. McDonald, K. & Müller-Reichert, T. Cryomethods for thin section electron microscopy. *Methods Enzymol.* **351**, 96-123 (2002).
18. Giddings, T. Freeze-substitution protocols for improved visualization of membranes in high-pressure frozen samples. *J. Microsc.* **212**, 53-61 (2003).

19. Walther, P. & Ziegler, A. Freeze substitution of high-pressure frozen samples: The visibility of biological membranes is improved when the substitution medium contains water. *J. Microsc.* **208**, 3-10 (2002).
20. Buser, C. & McDonald, K. Correlative GFP-immunoelectron microscopy in yeast. *Methods Enzymol.* **470**, 603-618 (2010).
21. Reynolds, E.S. The use of lead citrate at high pH as an electron-opaque stain in electron microscopy. *J. Cell Biol.* **17**, 208-212 (1963).
22. Proctor, R.H., Hohn, T.M. & McCormick, S.P. Restoration of wild-type virulence to Tri5 disruption mutants of *Gibberella zeae* via gene reversion and mutant complementation. *Microbiology* **143**, 2583-2591 (1997).
23. Chanda, A., Roze, L.V., Pastor, A., Frame, M.K. & Linz, J.E. Purification of a vesicle–vacuole fraction functionally linked to aflatoxin synthesis in *Aspergillus parasiticus*. *J. Microbiol. Methods* **78**, 28-33 (2009).
24. Wisniewski, J.R., Zougman, A., Nagaraj, N. & Mann, M. Universal sample preparation method for proteome analysis. *Nat. Methods* **6**, 359 (2009).
25. Elias, J.E. & Gygi, S.P. Target-Decoy Search Strategy for Mass Spectrometry-Based Proteomics, in *Proteome Bioinformatics*. (eds. S.J. Hubbard & A.R. Jones) 55-71 (Humana Press, Totowa, NJ; 2010).
26. Kim, S. & Pevzner, P.A. MS-GF+ makes progress towards a universal database search tool for proteomics. *Nat Commun* **5** (2014).
